# The Micro-Expression Decoder: A Preliminary Mixed-Methods Evaluation of Training in Subtle Facial and Non-Verbal Cue Recognition in Final-Year Medical Students

**DOI:** 10.3390/clinpract16090170

**Published:** 2026-09-14

**Authors:** Hashira Syed, Katerina Sini, Andreas Conte, Austen El-Osta, Azeem Majeed, Waseem Jerjes

**Affiliations:** 1 School of Public Health, Faculty of Medicine, Imperial College London, London SW7 2AZ, UKkaterina.sini22@imperial.ac.uk (K.S.); a.el-osta@imperial.ac.uk (A.E.-O.); a.majeed@imperial.ac.uk (A.M.); 2 Faculty of Life Sciences & Medicine, King’s College London, London SE1 1UL, UK; andreas.conte@me.com

**Keywords:** medical education, non-verbal communication, subtle facial cues, standardised patients, simulation, mixed-methods research, communication skills

## Abstract

**Background**/**Objectives:** Subtle facial and interactional cues may prompt clinicians to explore possible anxiety, confusion or hesitation, but they do not establish a patient’s internal state. The Micro-Expression Decoder (MED) links cue recognition with tentative interpretation, direct checking and communication adaptation. **Methods:** This preliminary, uncontrolled, single-group pre–post convergent mixed-methods evaluation involved 60 final-year medical students from three London medical schools. Ten groups of six completed a 270 min simulation-based programme comprising conceptual teaching, video observation, standardised-patient encounters, feedback and reflection. Study-specific self-ratings and a parallel-form recognition task were completed before teaching and immediately after the instructional and simulation components, before the final debrief. Standardised-patient ratings were obtained during the first and final simulated encounters. There was no control group or follow-up. **Results:** Recognition confidence increased from 2.7 to 4.3 (mean change 1.6, 95% CI 1.39–1.81; dz = 1.98), interpretation confidence from 3.0 to 4.5 (dz = 2.01), standardised-patient-rated attentiveness from 3.2 to 4.6 (dz = 2.02), and satisfaction from 3.5 to 4.8 (dz = 1.94). Anxiety recognition increased from 25/60 (41.7%) to 55/60 (91.7%), and confusion recognition from 29/60 (48.3%) to 52/60 (86.7%). Qualitative accounts described earlier noticing, clarification and checking, alongside difficulty distinguishing cues, distraction, cultural variation and over-interpretation. Nineteen students increased confidence without categorical improvement in anxiety or confusion recognition. **Conclusions:** MED was associated with immediate improvement across study-specific outcomes in simulation. The uncontrolled design, immediate assessment, investigator-developed measures, expectancy effects and ceiling in post-training satisfaction preclude conclusions about causality, retention, real-patient benefit, empathy or clinical competence.

## 1. Introduction

Effective clinical communication depends on more than the words used. Facial movement, gaze, silence, posture and vocal delivery can shape how concern, uncertainty or hesitation is expressed and understood [1,2,3,4,5,6,7]. A clinician who notices a change in expression or a pause may have an opportunity to slow down, clarify an explanation or ask about a concern that has not yet been stated. The cue itself, however, is not proof of a particular emotion. Its value lies in prompting respectful enquiry rather than confident inference [8,9].

Strict micro-expressions are brief, involuntary facial movements that occur over a fraction of a second [3,4,5]. Clinical encounters also contain broader subtle cues that are not micro-expressions in the technical sense, including uncertain gaze, hesitant nodding and pauses. The MED programme name was retained because it was used during development and delivery, but the intervention taught a wider construct: recognition of subtle facial and non-verbal cues, followed by contextual interpretation and direct checking. This distinction is important because the meaning of facial behaviour varies across individuals, situations and cultures [9,10,11,12,13,14].

Communication curricula usually cover consultation structure, active listening, explanation and empathy, with non-verbal behaviour often encountered indirectly through OSCE preparation and clinical placements [7,12,15,16,17,18]. More focused programmes have differed substantially in scope and assessment [19,20,21,22,23]. Molinuevo et al. described a curricular model for second-year students, including a theoretical framework, learning strategies and assessment tools, but not a controlled outcome evaluation [19]. Ragsdale et al. evaluated a 90 min workshop delivered at six venues to 156 healthcare providers and trainees, with immediate pre–post measures of skill, knowledge, perceived importance and confidence [8]. Yu et al. randomised 82 first- and second-year students to a one-hour METT/SETT class or repeated testing without the class and assessed immediate recognition-test performance [13]. Park and Park studied 51 first-year students before and after a broader communication course that included repeated standardised-patient practice and feedback, using video-coded non-verbal behaviours [11]. Kelly et al. evaluated drama workshops attended by 112 conference participants through narrative material and post-workshop open responses, with attention to sight, sound, touch and proxemics [6].

MED brought several of these elements together in one programme: cue recognition, application within a consultation, rating before feedback, explicit correction of over-interpretation and structured reflection. Its four-stage pathway—notice, interpret tentatively, check and adapt—treats recognition as the start of a communication process rather than as a stand-alone decoding skill. The present study does not show that this combined format is superior to any earlier programme. 

Mehrabian’s work is sometimes reduced to a universal percentage rule for verbal and non-verbal communication, although it concerned the communication of attitudes and feelings under constrained conditions [16]. MED did not assume a fixed proportion of meaning is conveyed non-verbally. Its narrower premise was that subtle cues may be clinically useful when they lead to careful checking rather than deterministic interpretation [24,25,26,27,28,29,30].

This study was designed as a preliminary, simulation-based educational evaluation. Its purpose was to describe programme delivery and examine immediate change in prespecified, study-specific outcomes across MED, not to establish effectiveness in real clinical practice. The two primary outcomes were self-rated confidence in recognising subtle facial cues and in interpreting their possible emotional significance. The six secondary outcomes comprised two standardised-patient-rated items, two recognition-task outcomes and two student questionnaire items. The qualitative strand explored how students and standardised patients understood the changes, including difficulties and reservations.

## 2. Materials and Methods

### 2.1. Study Design and Reporting Approach

This was a preliminary, uncontrolled, single-group pre–post convergent mixed-methods educational evaluation. Quantitative and qualitative material was collected during the same programme, analysed separately and integrated during interpretation through a joint display and participant-level examination of convergence and discordance. The quantitative strand assessed immediate change; the qualitative strand examined how participants experienced cue recognition and response. DoCTRINE was used as the overarching education-specific reporting framework [28]. STROBE informed relevant observational elements of the uncontrolled quantitative strand [24], TIDieR informed intervention description and reproducibility [25], and GRAMMS informed the rationale, timing, separate analyses and integration of the mixed-methods design [26]. Supplementary Materials S1–S23 provide detailed supporting information on the MED construct, outcome definitions, study instruments and scenarios, quantitative and qualitative findings, intervention fidelity, sensitivity analyses, participant-level patterns, comparisons with previous programmes, sample-size considerations and reporting-framework mapping. Supplementary Table S23 summarises how each reporting framework informed the study and where its main reporting elements are addressed. 

### 2.2. Setting, Participants, and Recruitment

Ten face-to-face sessions were delivered in university or medical-education simulation facilities in North London between 12 October 2023 and 13 November 2025. Two sessions were held in 2023, four in 2024 and four in 2025, with six students in each session. The verified dates were 12 October and 7 December 2023; 14 March, 20 June, 19 September and 21 November 2024; and 13 February, 15 May, 18 September and 13 November 2025. No NHS patients or clinical care were involved.

Final-year medical students from three London medical schools were invited through administrative mailing lists. The institutions are reported as Schools A–C. Administrators supplied the numbers invited but not the size of each potentially eligible final-year population. Students were eligible if they were in their final year, could attend the full session and had not previously completed formal micro-expression training. Interested students contacted the study team and received written information before consent. Investigators did not approach individual students directly. Participants received refreshments and a certificate of attendance, but no payment, academic credit or assessment advantage. Routine prior teaching included consultation structure, active listening, empathy, checking understanding, OSCE preparation and clinical placements; none reported previous formal training specifically in micro-expressions or systematic subtle-cue recognition.

### 2.3. MED Construct and Programme Development

MED distinguished strict micro-expressions—brief involuntary facial movements—from broader subtle facial and interactional cues. The assessed cues were lip compression, widened eyes, facial tension, raised eyebrows, hesitant nodding, uncertain gaze and a hesitant pause. The first four were treated as facial behaviours; nodding, gaze and pauses were taught as broader interactional cues. Posture and vocal tone were discussed as contextual information but were not scored as principal recognition outcomes. Students were repeatedly reminded that a cue could suggest several possible meanings and should never be treated as diagnostic evidence of emotion (Supplementary Table S1).

A multidisciplinary team of clinicians and medical educators developed the programme from literature on facial-expression recognition, non-verbal communication and standardised-patient teaching [8,11,13,19]. Two medical educators and one communication-skills specialist reviewed the cue taxonomy, scenarios and scoring rules. Cognitive piloting involved eight students and an intervention pilot involved six learners; neither group entered the final study. Piloting led to wording, timing and difficulty adjustments before data collection. After the first two study sessions, minor clarifications were made to facilitator instructions without changing participant materials, learning objectives, outcome definitions or scoring.

### 2.4. Intervention

Each session lasted 270 min, comprising 255 min of structured activity and a 15 min break. Four educators delivered the programme across the ten sessions: two general-practitioner medical educators, one simulation fellow and one communication-skills psychologist. Two educators attended each session, giving an educator-to-student ratio of 1:3; the same pair did not deliver every session. All used a common manual, slide deck, cue taxonomy, scenario pack, feedback rubric and fidelity checklist (Supplementary Instrument S18).

The teaching component used 14 acted clips, each approximately 5–20 s: eight obtained from a licensed educational video library and six recorded specifically for MED using study-developed scripts. These teaching clips were separate from the 14 clips used in the recognition assessment. The assessment comprised seven initial clips and seven parallel final clips, of which seven were obtained from licensed educational materials and seven were purpose-recorded for MED. No assessment clip was shown or discussed during teaching. Supplementary Table S5 provides the available item-level provenance and characteristics of the recognition-assessment stimuli, including study-level identifiers, observable cues, source category, target category, response key and parallel-form matching. The parallel assessment forms were matched prospectively by target category, observable cue, duration and intended intensity. Students also completed two eight-minute simulated encounters with different clinical content but matched anxiety and confusion/uncertainty cues (Supplementary Table S16). Table 1 presents the sequence and timing; operational details, study instruments and scenario summaries are provided in the Supplementary Materials (Supplementary Note S14). Licensed video files cannot be redistributed because of copyright restrictions; the available item-level provenance and study-archive identifiers are therefore reported in the Supplementary Materials to document the stimuli used in MED.

### 2.5. Standardised Patient Encounters and Recognition Task

Each student completed two parallel eight-minute simulated encounters with different clinical content and matched planned cues. Six standardised patients with prior undergraduate-simulation experience completed a two-hour MED-specific calibration session and a further 90 min rehearsal. Training covered scenario content, cue timing and intended intensity, rating-form use and avoidance of coaching during assessed encounters. Each student saw a different standardised patient in the first and final encounters. Allocation was balanced across sessions, and identifiers for both the first-encounter and final-encounter standardised patients were retained.

Complete blinding was not possible because standardised patients participated in the educational programme and could infer whether an encounter was the first or final assessment. They were not shown the student’s earlier scores and were instructed to rate only observed behaviour. The same standardised patient performed the scenario, completed the rating before providing feedback and then contributed educational feedback; no independent blinded assessor was used.

The student questionnaire, recognition task and standardised-patient form were investigator-developed and study-specific. Items were derived from the learning objectives, reviewed by clinicians and medical educators, cognitively piloted before data collection, and supported by a scoring guide agreed in advance. These procedures provide limited evidence relating to content and response process, but they do not establish internal structure, relationships with external variables or the consequences of score use [29,30]. A 2025 scoping review of communication-skills assessment similarly found that response-process and consequence evidence was rarely reported and recommended that authors define the construct, intended score interpretation and intended use explicitly [31]. Formal psychometric validation, test–retest reliability and external criterion validity were not established. The scores are therefore interpreted only as study-specific indicators and not as validated measures of clinical competence.

The original student questionnaire retained the term “micro-expressions” in several items. Those items are reproduced verbatim in Supplementary Instrument S11 and are interpreted as study-specific self-ratings rather than pure measures of the broader subtle-cue construct.

Students rated nine statements on five-point scales. The two prespecified primary outcomes were the single-item self-ratings of confidence in recognising subtle facial cues (Q1) and confidence in interpreting their possible emotional significance (Q2). Six prespecified secondary outcomes were standardised-patient-rated attentiveness (SP1), standardised-patient-rated satisfaction (SP8), anxiety recognition, confusion recognition, confidence using non-verbal communication (Q6) and perceived importance of cue recognition for rapport (Q7). Five additional student questionnaire items—Q3, Q4, Q5, Q8 and Q9—were analysed as exploratory outcomes, as were uncertainty recognition and five standardised-patient items (SP2-SP5 and SP7). SP6 is retained in the reproduced instrument and descriptive table to show the wording actually administered (Supplementary Instrument S12), but it was excluded from paired inference because the phrase “improved the quality” had no valid reference point at the first encounter (Supplementary Table S4). The complete outcome map is provided in Supplementary Table S2.

The recognition task contained seven clips at each time point: three anxiety-targeted clips, three confusion-targeted clips and one uncertainty-targeted clip. Initial and final forms used parallel rather than identical clips, and each clip was shown once. Each form therefore yielded a maximum raw score of 7, with one point available for each clip. Anxiety and confusion domain scores each ranged from 0 to 3, and the single uncertainty item was reported separately as an exploratory binary outcome. The 0–7 raw total was descriptive and was not a prespecified primary or secondary outcome. For each clip, students selected one cue category from anxiety, confusion, uncertainty, discomfort or unsure and one clinical response from the response options reproduced verbatim in Supplementary Instrument S15. A clip was scored as correct only when both selections matched the prespecified answer guide. Anxiety and confusion were classified as meeting the criterion when at least two of the three relevant clips were correct; uncertainty was reported for its single clip. The participant instructions and response options are reproduced in Supplementary Instrument S15; the response forms are shown with administrative clip codes included for documentation, and the prespecified answer key is provided in Supplementary Table S5.

For categorical survey analyses, scores of 4 or 5 were classified as meeting the criterion and scores of 1–3 as not meeting it. Improvement meant movement from not meeting to meeting the criterion, decline meant the reverse, and all within-category movements were classified as no categorical change. Continuous paired analyses of the questionnaire items were retained to avoid discarding within-category information (Supplementary Table S3).

### 2.6. Quantitative Analysis

Reanalysis of the de-identified participant-level dataset was undertaken in Python 3.13.5 (Python Software Foundation, Beaverton, OR, USA), using NumPy 2.3.5 and SciPy 1.17.0 (NumFOCUS-affiliated open-source projects, Austin, TX, USA). Five-point outcomes are reported as means with SDs and medians with interquartile ranges. Paired t-tests estimated mean change with 95% CIs; Wilcoxon signed-rank tests were used as ordinal sensitivity analyses. Cohen’s d_z was calculated as the mean individual change divided by the SD of individual change, with percentile-bootstrap 95% CIs from 20,000 participant-level resamples. The distribution of individual changes and proportions at the maximum score were examined explicitly.

Binary paired outcomes are reported as counts, percentages and transition matrices, with exact McNemar tests based on discordant pairs. No multiplicity adjustment was applied; primary, secondary and exploratory outcomes are distinguished and estimates are interpreted with their uncertainty. Exploratory clustering sensitivity analyses compared continuous change scores across school, session and final-encounter standardised patient using one-way analyses, and compared binary improvement proportions using chi-square tests (Supplementary Tables S8 and S9). These analyses were descriptive because there were only three schools, ten sessions and six assessors. Because different standardised patients rated the first and final encounters, stratification by the final-encounter assessor did not capture all possible first/final assessor-pair effects.

### 2.7. Qualitative Analysis and Mixed-Methods Integration

The qualitative corpus comprised 56 reflective journals, 60 post-training open responses, 60 standardised-patient comment forms and ten group-debrief records (Supplementary Instrument S13). A codebook form of thematic analysis, rather than reflexive thematic analysis, was used within a pragmatic contextualist orientation [27]. Coding combined deductive categories based on notice, interpret, check and adapt with inductive codes arising from the material (Supplementary Table S10). Frequencies refer to the number of distinct source records containing a theme, not the number of coded passages.

Two analysts documented their assumptions before coding. One was a clinician–medical educator who had helped develop MED and was familiar with the intended notice–interpret–check–adapt pathway; the second was an independent qualitative health researcher who had not contributed to programme design or delivery. The developer’s involvement created a risk that the deductive framework, code selection and emphasis of themes would favour the intended programme logic. The independent analyst’s double-coding of the prespecified sample, review of candidate-theme summaries and review of all negative or discrepant cases were used to reduce this risk, but could not remove it. Twelve of 56 journals, 12 of 60 post-training open responses, 12 of 60 standardised-patient comments and two of ten debrief records were independently double-coded, purposively spanning all ten groups and all three schools. The codebook was refined after comparison. One analyst coded the remaining material in NVivo 15 (Lumivero, Denver, CO, USA), and differences were resolved through documented discussion. Quotations are verbatim apart from removal of repeated words or obvious typographical errors, and are identified by an anonymous source.

The administered prompts were also reviewed for directionality before final interpretation. The pre-training question asking what participants hoped to gain, student post-training questions 2–4, standardised-patient open questions 1, 3 and 4, and reflective-journal prompts 1, 3–6 and 8–10 either primed an expected benefit, assumed that change had occurred or invited a favourable account. This wording could increase affirmative responses and narrow the range of criticism. Prompt source was therefore retained during coding, responses to these items were treated as elicited accounts rather than spontaneous confirmation of effectiveness, and negative, neutral and discrepant material was actively sought. Supplementary Table S22 reproduces the relevant wording and states how each prompt was handled.

Integration occurred after separate analyses. A joint display compared quantitative results with qualitative explanations and reservations. Participant-level linkage was used to identify increases in either primary confidence item without corresponding categorical improvement in anxiety or confusion recognition. Convergence was treated as supportive context rather than independent confirmation because all strands were collected close to the same intervention.

### 2.8. Sample Size Consideration

The original a priori calculation was performed in G*Power 3.1.9.7 (Heinrich Heine University Düsseldorf, Düsseldorf, Germany) for a two-sided paired-samples t-test with alpha = 0.05, 80% power and a conventional moderate paired effect of dz = 0.50. Because dz standardises the mean paired difference by the SD of the paired differences, no separate pre–post correlation is entered in this parameterisation. The resulting minimum was 34 independent participants. The planning effect was not estimated from previous MED data, no separate attrition allowance was prespecified, and the calculation did not adjust for the two primary outcomes or for clustering.

A planning sensitivity analysis was added rather than calculating power from the observed effect. Applying alpha = 0.025 for two primary outcomes increased the independent-participant requirement to 41. Using the design-effect formula 1 + (m-1)ICC, the illustrative requirement at an ICC of 0.05 was 51 participants for teaching sessions of six, 59 for final-encounter standardised-patient clusters averaging ten, and 80 for schools averaging 20 participants. Results across ICCs from 0.01 to 0.10 are shown in Supplementary Table S21. These are one-factor illustrations rather than a formal multilevel power calculation: clustering by teaching session, medical school and final-encounter standardised patient was considered separately; standardised patients were crossed with sessions, different assessors were used at the two encounters and reliable planning ICCs were not available. The achieved sample of 60 therefore does not ensure adequate power under every plausible clustering structure, particularly where school-level clustering is moderate.

### 2.9. Ethics and Governance

The project comprised a formative, simulation-based educational programme and a voluntary evaluation of its immediate educational outcomes. It was delivered in university or medical-education simulation facilities, was not part of summative assessment and had no bearing on marks, placements or academic progression. It did not take place in NHS clinical services or involve NHS patients, service users, clinical care, patient records, human tissue or confidential patient information. Under the national Health Research Authority framework, NHS organisational approval and NHS Research Ethics Committee review are considered separately. As the project was conducted outside NHS services, NHS organisational approval was not applicable. Before recruitment, the HRA decision tool was completed on 10 August 2023, more than two months before the first session on 12 October 2023, and it was concluded that NHS REC review was not required for sites in England. 

The local governance arrangements reflected the formative educational setting and the low-risk nature of the evaluation. Participation in the evaluation was voluntary and separate from programme attendance and academic assessment. Written student consent covered matched pre–post analysis, questionnaires and reflective journals, publication of aggregated findings and anonymised quotations, and withdrawal until anonymisation. Standardised patients consented to the analysis and anonymous quotation of their ratings and comments. Recruitment and consent were undertaken by administrators independent of academic assessment. Investigators did not examine participants or contribute to progression decisions, and educators had no access to identifiable responses. The evaluation involved only study-specific educational measures and reflective material; no sensitive clinical information was collected and no encounter was audio- or video-recorded.

Participants were assigned study identifiers. The encrypted linking file was held separately from the de-identified dataset on an institutionally approved secure server, with access limited to named investigators. Paper forms were kept in locked storage and digital records were password-protected. Data will be retained for 10 years after publication and then securely destroyed.

## 3. Results

### 3.1. Participant Flow and Cohort Characteristics

**Figure 1:**
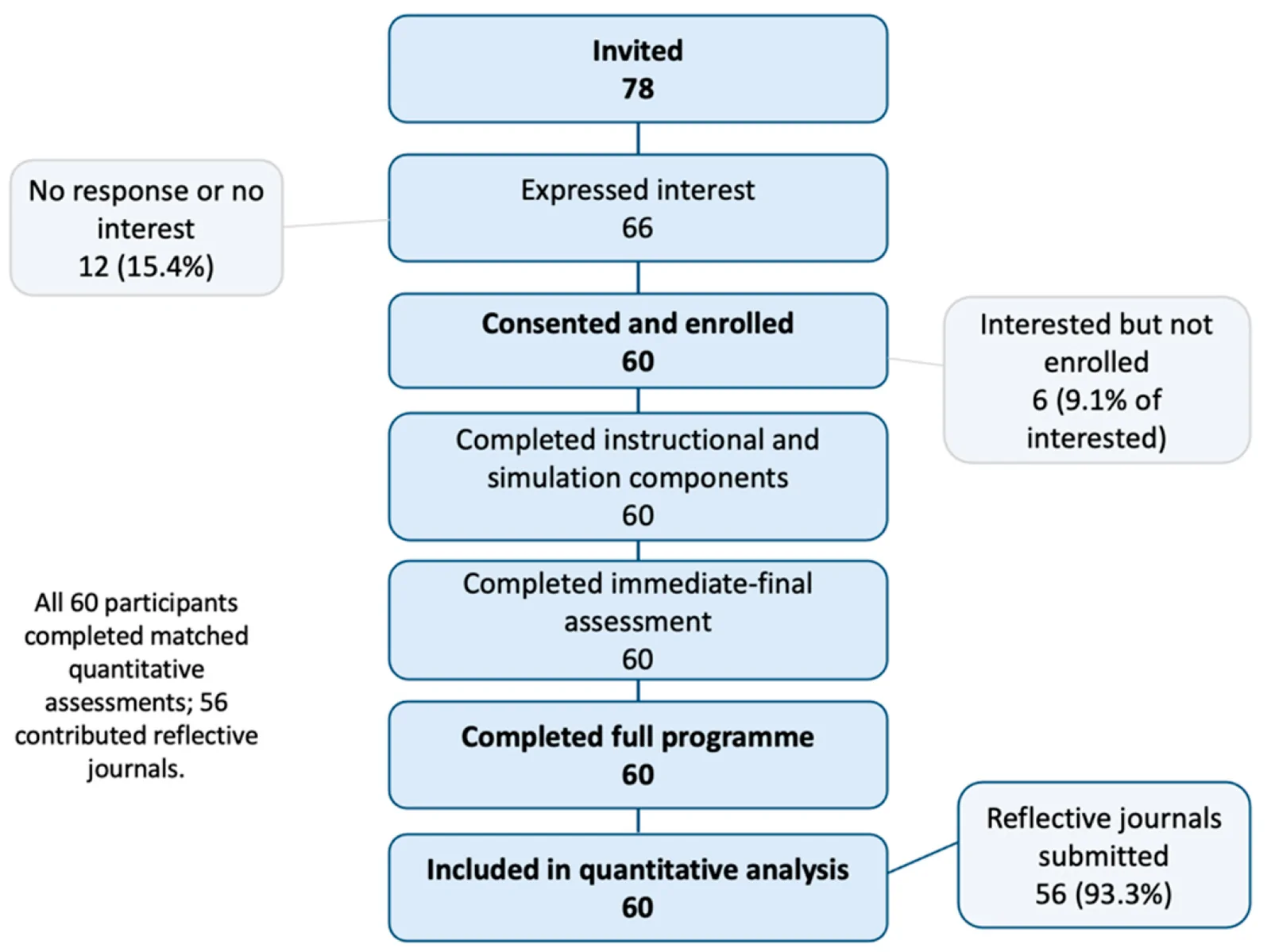
Participant flow.

Seventy-eight students were invited, 66 expressed interest and 60 enrolled. Six interested students did not enrol because of scheduling or placement commitments. No participant withdrew after enrolment. All 60 completed the intervention and matched quantitative assessments; 56 submitted reflective journals. The four non-submitters completed all quantitative measures. Their change scores did not show a consistent pattern of lower response, although the number was too small for meaningful comparison (Figure 1). Participant recruitment and cohort characteristics are summarised in Table 2, including school-level invitation, enrolment and completion numbers, demographic characteristics, prior cue-recognition training and clinical placement exposure.

**Table 2:** Recruitment by school and cohort characteristics.

Characteristic	School A	School B	School C	Total
Invited	28	26	24	78
Expressed interest	24	22	20	66
Enrolled	22	20	18	60
Completed quantitative assessment	22	20	18	60
Submitted reflective journal	—	—	—	56/60 (93.3%)
Age, years	—	—	—	Median 24 (IQR 23–25)
Gender	—	—	—	38 female; 22 male
Prior formal cue-recognition training	—	—	—	0/60
Clinical placement exposure	—	—	—	GP 60; medicine 52; surgery 48; paediatrics 36; psychiatry 28

Placement categories were not mutually exclusive. The potentially eligible final-year population at each school was not recorded.

### 3.2. Intervention Delivery and Fidelity

All ten sessions used the same intervention manual, participant materials, outcome definitions and scoring rules. Independent observers completed a 17-item checklist covering learning objectives, prescribed materials, simulations, feedback domains, debrief content and assessment timing (Supplementary Instrument S17). Mean fidelity was 93.5% (rounded to 94%), with session scores from 88.2% to 100% (Supplementary Table S7). Two trained observers shared the ten sessions.

### 3.3. Quantitative Initial-to-Final Outcomes

**Figure 2:**
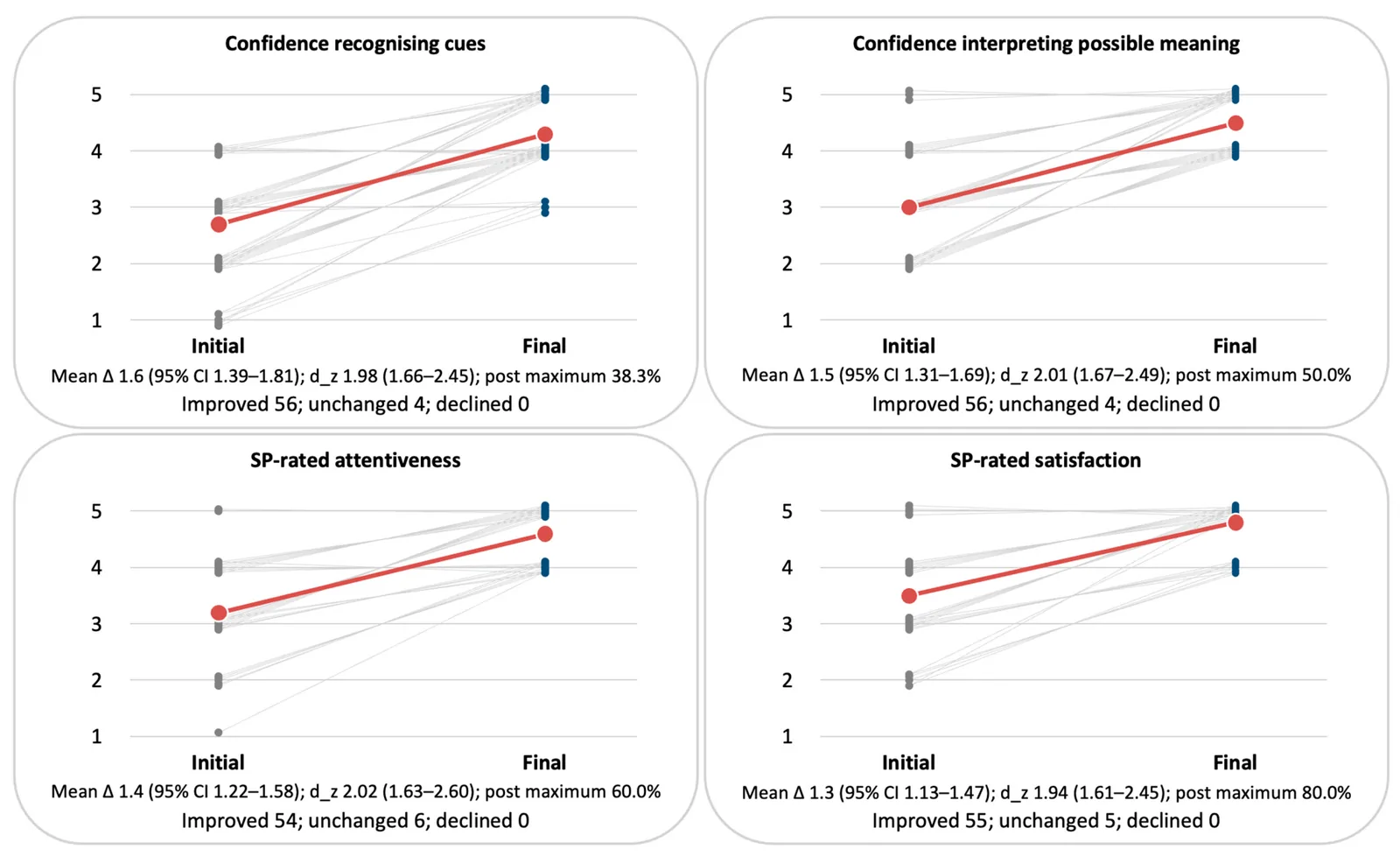
Paired initial and immediate final scores for the two primary confidence outcomes and two secondary standardised-patient-rated outcomes. Each line represents one participant. The timing of the initial measurement differed by outcome and is defined in the Table 3 footnote. Grey and blue dots represent individual participants’ initial and final scores, respectively; light-grey lines connect paired participant scores, and red dots and lines indicate the group means.

Both primary confidence outcomes and both secondary standardised-patient-rated outcomes increased between the initial and immediate final assessments (Table 3; Figure 2). Recognition confidence increased by a mean of 1.6 points (95% CI 1.39–1.81), and interpretation confidence by 1.5 points (95% CI 1.31–1.69). Standardised-patient-rated attentiveness increased by 1.4 points (95% CI 1.22–1.58), and standardised-patient-rated satisfaction by 1.3 points (95% CI 1.13–1.47). Paired t-tests and Wilcoxon sensitivity analyses were consistent (all *p* < 0.001).

The standardised changes were unusually large, with d_z values from 1.94 to 2.02. The participant-level distributions explain this pattern: no participant declined on any of the four principal continuous outcomes; four to six were unchanged and the remainder increased (Supplementary Figure S1). Ceiling was especially marked for standardised-patient-rated satisfaction, for which 48/60 (80.0%) received the maximum post-training score.

### 3.4. Categorical Learning Indicators and Recognition Performance

**Figure 3:**
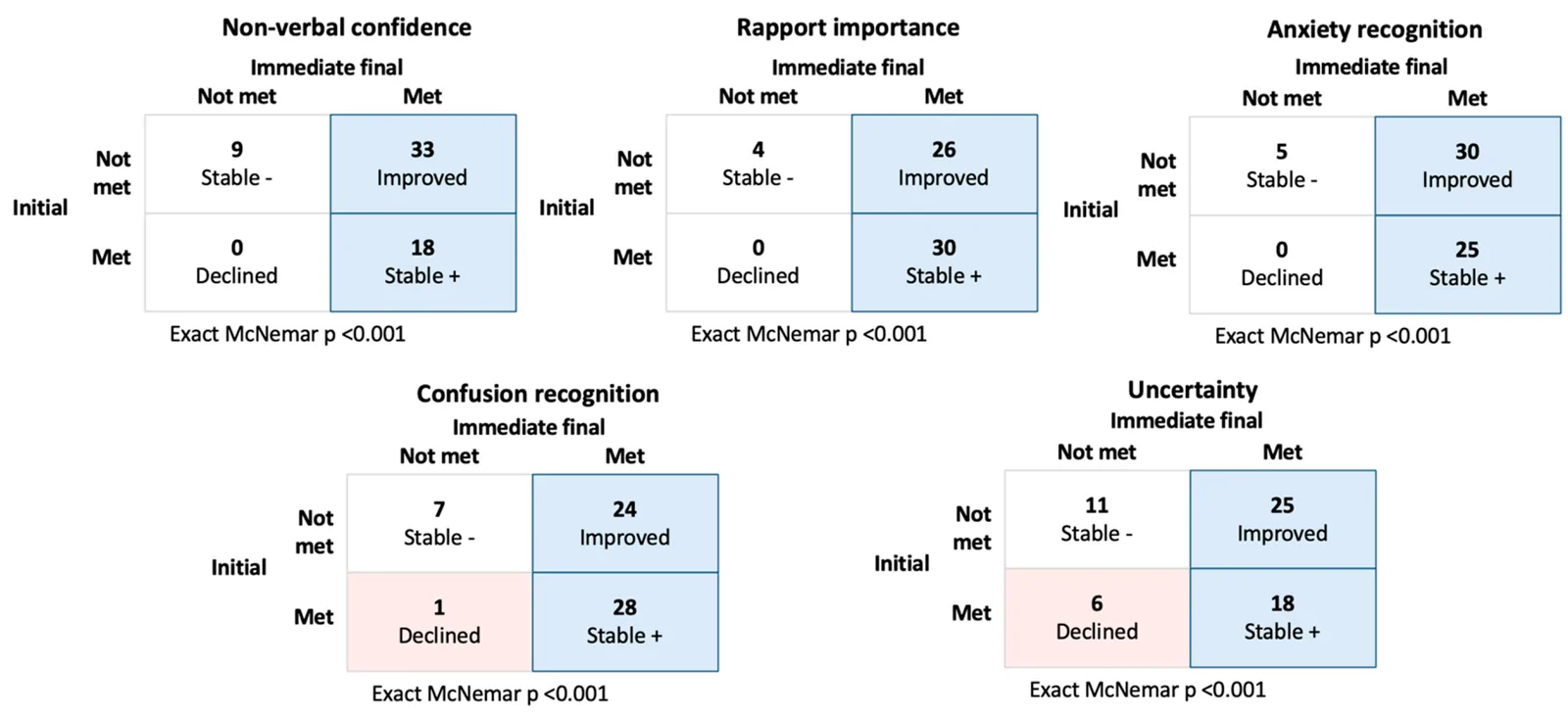
Paired categorical transitions for four secondary outcomes and one exploratory outcome. Rows indicate initial status and columns indicate immediate final status. Uncertainty recognition was exploratory. Blue shading indicates participants who met the criterion at the immediate final assessment (either improved or remained meeting the criterion), while pink shading indicates participants who declined from meeting to not meeting the criterion.

At the immediate final assessment, the proportion meeting the criterion increased for both secondary questionnaire indicators and both secondary recognition outcomes; the exploratory uncertainty item changed in the same direction (Table 4). The largest absolute changes were 55.0 percentage points for confidence using non-verbal communication and 50.0 percentage points for anxiety recognition. Transition matrices showed that improvement was distributed across the cohort: no declines occurred for the two questionnaire indicators or anxiety recognition, one participant declined in confusion recognition, and six declined on the exploratory uncertainty item (Figure 3; Supplementary Table S6).

### 3.5. Qualitative Findings

Six recurrent themes described how students and standardised patients experienced the programme (Table 5). Students commonly described noticing a possible cue earlier, slowing the encounter, checking rather than assuming, and changing an explanation. Standardised patients described greater attentiveness and responsiveness within the simulation. These accounts concern the immediate educational encounter and intended future use; they are not evidence of behaviour with real patients.

Less-positive accounts qualified the dominant themes. One student wrote, “At first I could not tell whether the patient was anxious or simply confused, so I had to ask rather than rely on the expression” (Student 19, reflective journal). Another recognised over-interpretation: “I realised I had read too much into one pause. The safer approach was to acknowledge it and check what it meant” (Student 33, post-training open response). Deliberate monitoring could also compete with history-taking: “Concentrating on facial cues sometimes distracted me from the history, particularly when I was trying to structure the consultation” (Student 47, reflective journal). Cultural variation was raised explicitly (Student 05, reflective journal), one student remained unsure about reliable recognition in a busy clinic (Student 58, post-training open response), and one standardised patient reported that a hesitant pause was initially missed (SP 6, post-training comment).

### 3.6. Mixed-Methods Integration

**Figure 4:**
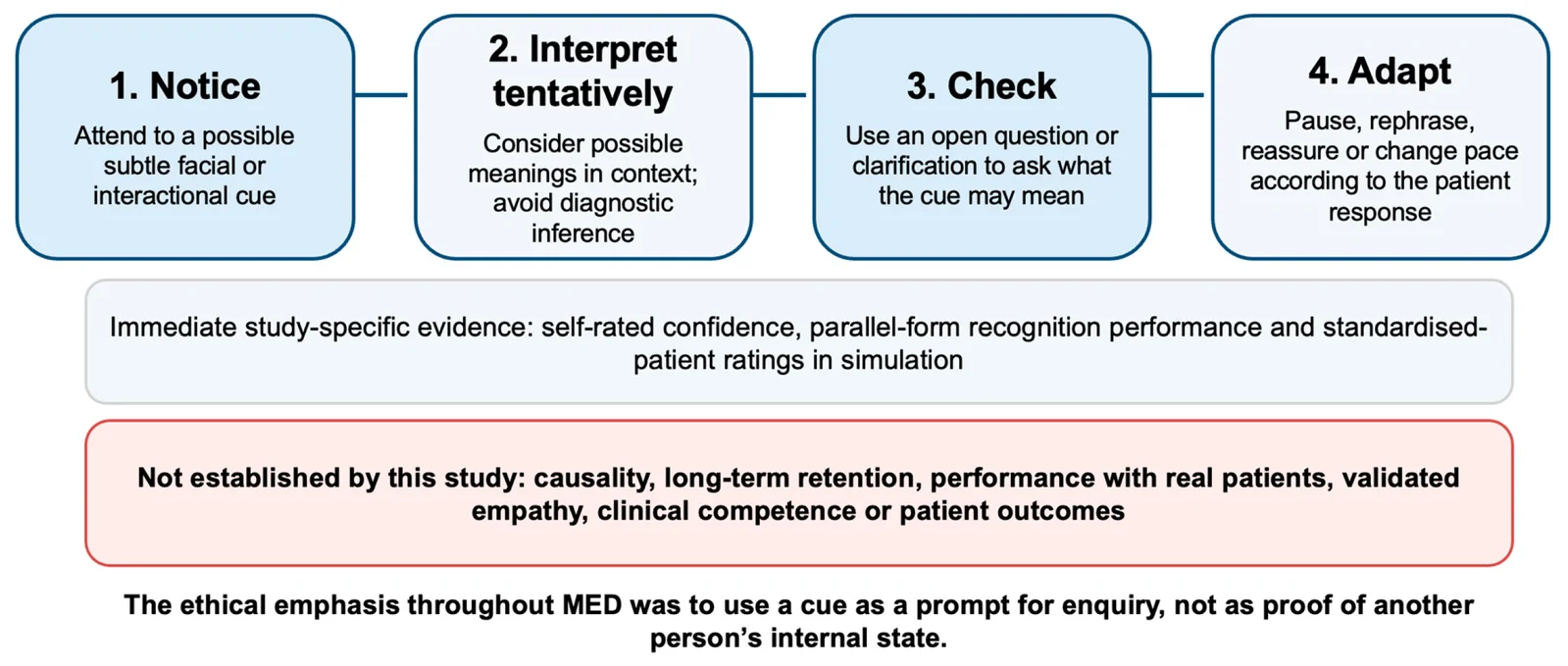
Proposed MED educational pathway. Conceptual interpretation only; the pathway was not tested as a causal or mediational model.

The joint display showed a coherent but not uniformly convergent account (Table 6). Numerical gains in anxiety and confusion recognition were reflected in descriptions of pausing, rephrasing and asking open questions. Participant-level linkage showed that 19/60 students increased on at least one primary confidence item without categorical improvement in either anxiety or confusion recognition (Supplementary Table S19). This discordance reinforces that confidence and task performance were not equivalent. Qualitative reservations further showed that increased awareness did not remove uncertainty, competing attentional demands or cultural ambiguity. The proposed MED educational pathway, together with the distinction between immediate study-specific evidence and outcomes not established by this study, is summarised in Figure 4.

## 4. Discussion

In this preliminary uncontrolled evaluation, MED was associated with immediate improvement in final-year medical students’ self-rated confidence, performance on a study-specific parallel-form recognition task and standardised-patient ratings during simulation. The qualitative material helped explain how students used the intended pathway—notice, interpret tentatively, check and adapt—but also documented uncertainty, over-interpretation, distraction and cultural variation. The findings therefore support the deliverability of MED and immediate educational responsiveness within this simulation, not clinical effectiveness.

Yu et al. provide the closest controlled comparison. They randomised 82 first- and second-year medical students, 41 per group, to a one-hour METT/SETT class or repeated testing without the class. Mean METT and SETT scores in the training group increased by 29.3% and 36.2%, respectively, while the control group showed an 11.0% increase on METT at repeat testing [13]. Their design therefore offers stronger evidence that the class altered immediate recognition-test performance. MED involved final-year students, lasted 270 min, used an uncontrolled single-group design and combined self-ratings, a parallel-form recognition task, standardised-patient ratings and qualitative material. The outcomes and designs are not directly commensurable: Yu et al. did not report a standardised effect that can be compared with MED’s within-group dz values, and MED’s larger numerical dz estimates should not be interpreted as evidence of superiority. Neither study established longer-term retention or benefit in real-patient consultations.

Beyond Yu et al., MED draws on facial-expression workshops, non-verbal communication curricula, standardised-patient feedback and drama-based approaches [6,8,11,19]. A structured comparison of MED with selected earlier programmes, including learner groups, programme content and duration, assessment approaches, standardised-patient involvement, reflection and follow-up, is provided in Supplementary Table S20. Its distinctive feature is not a claim that emotions can be read reliably from the face. It links noticing to a cautious consultation response, using rating before feedback, explicit correction of over-interpretation and structured reflection to move learners from detection towards enquiry. Whether this combination adds value beyond a shorter recognition workshop or a conventional communication course cannot be determined from the present design.

The magnitude and uniformity of the continuous changes require caution. The four d_z estimates were close to 2.0, no participant declined on any principal continuous outcome, and 80% received the maximum post-training satisfaction score. The participant-level calculations confirm that the values were not arithmetic errors: the SDs of individual change were small because most students increased by one or two scale points and none decreased. Nevertheless, the pattern is compatible with several non-specific influences, including immediate testing, demand characteristics, educator enthusiasm, heightened attention, social desirability and unblinded standardised-patient ratings. Ceiling effects also limit discrimination at the upper end. These effect sizes should not be compared directly with estimates from validated competence measures or interpreted as durable treatment effects.

Confidence and competence must remain separate. Confidence was prespecified as the primary educational outcome and is relevant to learner readiness, but it is especially responsive to an intensive, positively framed session. The recognition task offered a performance-based measure, yet it was investigator-developed and used parallel acted stimuli rather than an externally validated criterion. The participant-level discordance—19 students reporting greater confidence without categorical improvement in anxiety or confusion recognition—reinforces the need to assess both constructs. Future studies should use validated or independently benchmarked tasks and consider placing performance outcomes ahead of confidence.

Recent assessment work also points towards a more independent, multimethod approach. Zerbini et al. studied 43 medical students in simulated consultations using blinded expert and standardised-patient ratings alongside video-derived non-verbal measures [32]. Their study was cross-sectional rather than an intervention trial, but the separation of performer, assessor and measurement source offers a useful model for a future controlled evaluation of MED.

The qualitative findings provide context rather than independent proof. One analyst had helped develop MED, participants knew the educational purpose, and several post-training, reflective-journal and standardised-patient prompts explicitly presupposed change, benefit or successful application. These features create a material risk of confirmation and social-desirability bias. Independent double-coding, review of candidate themes and review of negative or discrepant cases reduce but do not remove that risk. The findings should therefore be read as accounts elicited within the programme, not as neutral confirmation that MED was effective. The report that cue monitoring could distract from history-taking is particularly important: adding an observational task may increase cognitive load, especially for less experienced learners. The emphasis on asking rather than assuming, and on cultural and individual variation, should remain central to any future use of MED.

MED’s use of simulation, structured feedback and reflection is consistent with broader work on translating communication principles into observable practice and integrating theory with clinical education [21,22,23]. The present design, however, cannot identify which component produced the observed change.

Several design limitations constrain inference. There was no control group, randomisation or repeated follow-up, so change cannot be separated from practice effects, task familiarity, Hawthorne effects or expectancy. Outcomes were measured immediately after one session and do not establish retention or transfer to real consultations. Participants self-selected and may have been unusually interested in communication. The study-specific measures lacked formal psychometric validation, and SP6 contained comparative wording that made it unsuitable for paired inference. Standardised patients were not blinded, combined performer, assessor and feedback roles, and could infer assessment timing. One qualitative analyst had helped develop MED, and several prompts were directional. The original sample-size calculation assumed independent observations and did not adjust for two primary outcomes; the added sensitivity analysis shows that 60 participants may be insufficient under moderate school-level clustering. Different standardised patients rated the first and final encounters, so assessor-pair effects were not fully captured. The parallel recognition forms were matched operationally rather than psychometrically equated. The first standardised-patient rating followed the conceptual workshop and video activity and therefore was not a true pre-intervention baseline. Multiple outcomes were tested without multiplicity adjustment, post-training ceiling was substantial, and the inclusion of final-year students from three London schools limits transferability.

A controlled next-stage evaluation should use an attention-matched comparison group, independent blinded assessment where feasible, externally validated or benchmarked recognition measures, and follow-up after several weeks or months. Assessment in real clinical settings would need patient consent and appropriate governance, and should focus on observable communication behaviours rather than claims that an internal emotional state has been decoded. It would also be useful to test whether the four-stage pathway can be taught with fewer cues and lower cognitive load, and whether effects differ by prior communication experience or cultural context.

## 5. Conclusions

In this uncontrolled single-group evaluation, MED was associated with higher scores at the immediate final assessment for final-year medical students’ self-rated confidence, a study-specific recognition task, and standardised-patient-rated attentiveness and satisfaction during simulated encounters. The programme’s most defensible educational contribution is its four-stage pathway: notice a possible cue, interpret it tentatively, check its meaning and adapt communication. The study does not establish causality, retention, real-patient benefit, empathy or clinical competence. Controlled studies using validated measures, independent assessment, longer follow-up and real clinical settings are required.

## Figures and Tables

**Table 1:** MED session sequence and standardisation.

Component	Time	Content	Standardisation
Consent and initial student measures	20 min	Written consent; initial student survey and recognition task completed before teaching	Common forms and prespecified scoring guide
Conceptual workshop	35 min	Definitions; ethical use of cues; notice–interpret–check–adapt pathway	Shared slide deck and facilitator guide
Video observation and cue labelling	40 min	Teaching clips; individual labelling and group discussion	Same teaching clips and sequence for all groups; assessment clips were not used in teaching
First simulated encounter (pre-practice)	15 min	Eight-minute encounter; SP rating completed before brief formative feedback	Parallel scenario; common rating form; different SP used at the final encounter
Structured teaching and paired practice	55 min	Cue recognition, tentative interpretation, open checking and communication adaptation	Two educators; common practice prompts
Scheduled break	15 min	Break	Included in total programme duration
Final simulated encounter and feedback	45 min	Parallel encounter; SP rating completed before structured SP and educator feedback	Different SP from first encounter; common rating form and feedback rubric
Final recognition task and survey	25 min	Parallel-form recognition task and immediate final survey	Prespecified scoring thresholds
Debrief and reflective writing	20 min	Description–analysis–application debrief and journal completion	Standard debrief template

Total programme duration was 270 min, comprising 255 min of structured activity and a 15 min scheduled break. The first standardised-patient encounter followed the conceptual and video components but preceded structured paired practice.

**Table 3:** Primary confidence outcomes and secondary standardised-patient-rated outcomes.

Outcome/Status	Initial: Mean ± SD; Median [IQR]	Immediate Final: Mean ± SD; Median [IQR]	Mean Change (95% CI); SDΔ	Paired *t*-Test	Wilcoxon	d_z (Bootstrap 95% CI)	Improved/Unchanged/Declined	Maximum Score Initial/Final
Recognition confidence/Primary	2.7 ± 0.9; 3.0 [2,3]	4.3 ± 0.6; 4.0 [4,5]	1.6 (1.39–1.81); SDΔ 0.81	t(59) = 15.36; *p* < 0.001	W = 0; *p* < 0.001	1.98 (1.66–2.45)	56/4/0	0.0%/38.3%
Interpretation confidence/Primary	3.0 ± 0.8; 3.0 [2,3]	4.5 ± 0.5; 4.5 [4,5]	1.5 (1.31–1.69); SDΔ 0.75	t(59) = 15.54; *p* < 0.001	W = 0; *p* < 0.001	2.01 (1.67–2.49)	56/4/0	5.0%/50.0%
SP-rated attentiveness/Secondary	3.2 ± 0.7; 3.0 [3,4]	4.6 ± 0.5; 5.0 [4,5]	1.4 (1.22–1.58); SDΔ 0.69	t(59) = 15.63; *p* < 0.001	W = 0; *p* < 0.001	2.02 (1.63–2.60)	54/6/0	3.3%/60.0%
SP-rated satisfaction/Secondary	3.5 ± 0.8; 4.0 [3,4]	4.8 ± 0.4; 5.0 [5–5]	1.3 (1.13–1.47); SDΔ 0.67	t(59) = 15.00; *p* < 0.001	W = 0; *p* < 0.001	1.94 (1.61–2.45)	55/5/0	8.3%/80.0%

For Q1 and Q2, the initial assessment occurred before any teaching, and the immediate-final assessment occurred after the instructional and simulation components but before the final debrief. For SP1 and SP8, ratings were obtained after the first and final simulated encounters; their exact timing is shown in Table 1. All scales ranged from 1 to 5. Cohen’s dz was calculated as the mean individual change divided by the SD of individual change. Bootstrap confidence intervals used 20,000 participant-level resamples.

**Table 4:** Secondary and exploratory categorical outcomes.

Status	Outcome	Initial	Immediate Final	Absolute Change	Exact McNemar *p*
Secondary	Confidence using non-verbal communication	18/60 (30.0%)	51/60 (85.0%)	55.0 pp	<0.001
Secondary	Importance of cue recognition for rapport	30/60 (50.0%)	56/60 (93.3%)	43.3 pp	<0.001
Secondary	Anxiety recognition	25/60 (41.7%)	55/60 (91.7%)	50.0 pp	<0.001
Secondary	Confusion recognition	29/60 (48.3%)	52/60 (86.7%)	38.3 pp	<0.001
Exploratory	Uncertainty recognition	24/60 (40.0%)	43/60 (71.7%)	31.7 pp	<0.001

For student questionnaire items, scores of 4–5 met the criterion and scores of 1–3 did not. Anxiety and confusion recognition required at least two of three clips to have both the correct category and an acceptable clinical response. Uncertainty recognition was based on one exploratory clip. pp, percentage points.

**Table 5:** Core qualitative themes and illustrative extracts.

Theme	Frequency/Source	Illustrative Extract	Anonymous Source
Recognition of possible anxiety	Journals: 39/56; SP comments: 22/60	“I noticed the patient looked anxious when they pressed their lips together, which helped me address their concerns.”	Student 14, reflective journal
Clarification in response to confusion	Journals: 35/56; SP comments: 19/60	“The patient’s raised eyebrows made me realise they did not understand my explanation, so I rephrased it.”	Student 27, reflective journal
Checking rather than assuming	Journals: 42/56; debrief groups: 9/10	“It reminded me to check what the patient was feeling instead of deciding for them.”	Student 41, post-training open response
Enhanced engagement and presence	Journals: 40/56; SP comments: 45/60	“The student noticed my nervousness and paused to ask how I was feeling. It made the interaction feel more personal.”	SP 3, post-training comment
Reflective growth	Journals: 31/56	“I used to miss these subtle signs completely. Now I actively look for them.”	Student 08, reflective journal
Intended transfer to future practice	Journals: 28/56; debrief groups: 8/10	“I plan to look for these cues during sensitive conversations.”	Student 52, reflective journal

**Table 6:** Mixed-methods joint display linking quantitative outcomes to qualitative interpretation.

Domain	Quantitative Result	Qualitative Context	Discordance or Interpretive Limit
Primary confidence outcomes	Mean changes: Q1 +1.6; Q2 +1.5	Students described deliberate noticing and reflective growth	Nineteen students increased on at least one primary confidence item without categorical improvement in anxiety or confusion recognition; confidence was not equivalent to task performance
Anxiety recognition	25/60 to 55/60	Students described lip compression, widened eyes and facial tension as prompts to enquire about possible concern	Some students initially found anxiety difficult to distinguish from confusion
Confusion recognition	29/60 to 52/60	Students described rephrasing and checking understanding	One participant declined categorically; parallel forms and immediate testing do not eliminate practice effects or familiarity with the task format
SP-rated attentiveness and satisfaction	Mean changes +1.4 and +1.3	SPs described students as more observant and responsive	Assessors were unblinded, also provided feedback and showed marked final-score ceiling
Intended future use	Not assessed quantitatively	Students described settings in which cue awareness might be useful	No follow-up or real-patient observation was undertaken

## Data Availability

The de-identified participant-level data and analysis code supporting the reported results are available from the corresponding author on reasonable request, subject to confirmation that the proposed use is consistent with participant consent and applicable data-protection requirements. Licensed video clips cannot be redistributed; their operational characteristics are described in the Supplementary Materials.

## Supplementary Materials

The following supporting information can be downloaded at: https://www.mdpi.com/article/10.3390/clinpract16090170/s1, Table S1: Operational cue taxonomy and construct mapping; Table S2: Outcome map and analysis plan; Table S3: Results for all nine student questionnaire items; Table S4: Results for all eight standardised-patient items; Table S5: Recognition-task clip map and prespecified answer guide; Table S6: Underlying categorical transition counts; Table S7: Fidelity by session; Table S8: Continuous-outcome clustering sensitivity analyses; Table S9: Binary-improvement clustering sensitivity analyses; Table S10: Qualitative coding framework and illustrative positive and discrepant extracts; Instrument S11: Student evaluation form; Instrument S12: Standardised-patient feedback form; Instrument S13: Reflective journal prompts; Note S14: Administrative and analytic details; Instrument S15: Recognition-task response forms; Table S16: Simulated-encounter scenario and cue map; Instrument S17: Seventeen-item fidelity checklist; Instrument S18: Feedback rubric and debrief structure; Table S19: Participant-level confidence and recognition discordance; Table S20: Comparison of MED with selected earlier programmes; Table S21: Illustrative effect of multiplicity and clustering on the planned sample; Table S22: Directionality of administered qualitative prompts and analytic handling; Table S23: Reporting-framework map; Figure S1: Individual change scores.

## References

[1] Chichirez C.M., Purcărea V.L. (2018). Interpersonal communication in healthcare. J. Med. Life.

[2] Boissy A., Windover A.K., Bokar D., Karafa M., Neuendorf K., Frankel R.M., Merlino J., Rothberg M.B. (2016). Communication Skills Training for Physicians Improves Patient Satisfaction. J. Gen. Intern. Med..

[3] Shen X., Chen W., Zhao G., Hu P. (2019). Editorial: Recognizing Microexpression: An Interdisciplinary Perspective. Front. Psychol..

[4] Ekman P. (1992). Facial expressions of emotion: An old controversy and new findings. Philos. Trans. R. Soc. B.

[5] Dong Z., Wang G., Lu S., Li J., Yan W., Wang S.J. (2022). Spontaneous Facial Expressions and Micro-expressions Coding: From Brain to Face. Front. Psychol..

[6] Kelly M., Nixon L., Broadfoot K., Hofmeister M., Dornan T. (2019). Drama to promote non-verbal communication skills. Clin. Teach..

[7] Ranjan P., Kumari A., Chakrawarty A. (2015). How can Doctors Improve their Communication Skills?. J. Clin. Diagn. Res..

[8] Ragsdale J.W., Van Deusen R., Rubio D., Spagnoletti C. (2016). Recognizing Patients’ Emotions: Teaching Health Care Providers to Interpret Facial Expressions. Acad. Med..

[9] Hall J.A. (2011). Clinicians’ accuracy in perceiving patients: Its relevance for clinical practice and a narrative review of methods and correlates. Patient Educ. Couns..

[10] Wu Q., Peng K., Xie Y., Lai Y., Liu X., Zhao Z. (2022). An ingroup disadvantage in recognizing micro-expressions. Front. Psychol..

[11] Park K.H., Park S.G. (2018). The effect of communication training using standardized patients on nonverbal behaviors in medical students. Korean J. Med. Educ..

[12] Kienle R., Freytag J., Lück S., Eberz P., Langenbeck S., Sehy V., Hitzblech T. (2021). Communication skills training in undergraduate medical education at Charité—Universitätsmedizin Berlin. GMS J. Med. Educ..

[13] Yu E.H., Choi E.J., Lee S.Y., Im S.J., Yune S.J., Baek S.Y. (2016). Effects of micro- and subtle-expression reading skill training in medical students: A randomized trial. Patient Educ. Couns..

[14] Stevenson F. (2014). Achieving visibility? Use of non-verbal communication in interactions between patients and pharmacists who do not share a common language. Sociol. Health Illn..

[15] Roter D.L., Frankel R.M., Hall J.A., Sluyter D. (2006). The expression of emotion through nonverbal behavior in medical visits. Mechanisms and outcomes. J. Gen. Intern. Med..

[16] Mehrabian A. (1981). Silent Messages: Implicit Communication of Emotions and Attitudes.

[17] Vogel D., Meyer M., Harendza S. (2018). Verbal and non-verbal communication skills including empathy during history taking of undergraduate medical students. BMC Med. Educ..

[18] Baugh A.D., Vanderbilt A.A., Baugh R.F. (2020). Communication training is inadequate: The role of deception, non-verbal communication, and cultural proficiency. Med. Educ. Online.

[19] Molinuevo B., Escorihuela R.M., Fernández-Teruel A., Tobeña A., Torrubia R. (2011). How we train undergraduate medical students in decoding patients’ nonverbal clues. Med. Teach..

[20] Pollux P.M. (2016). Improved categorization of subtle facial expressions modulates Late Positive Potential. Neuroscience.

[21] Sanson-Fisher R., Hobden B., Waller A., Dodd N., Boyd L. (2018). Methodological quality of teaching communication skills to undergraduate medical students: A mapping review. BMC Med. Educ..

[22] Bandiera G., Kuper A., Mylopoulos M., Whitehead C., Ruetalo M., Kulasegaram K., Woods N.N. (2018). Back from basics: Integration of science and practice in medical education. Med. Educ..

[23] Oudenampsen J., van de Pol M., Blijlevens N., Das E. (2023). Interdisciplinary education affects student learning: A focus group study. BMC Med. Educ..

[24] von Elm E., Altman D.G., Egger M., Pocock S.J., Gøtzsche P.C., Vandenbroucke J.P., STROBE Initiative (2007). The Strengthening the Reporting of Observational Studies in Epidemiology (STROBE) statement: Guidelines for reporting observational studies. PLoS Med..

[25] Hoffmann T.C., Glasziou P.P., Boutron I., Milne R., Perera R., Moher D., Altman D.G., Barbour V., Macdonald H., Johnston M. (2014). Better reporting of interventions: Template for intervention description and replication (TIDieR) checklist and guide. BMJ.

[26] O’Cathain A., Murphy E., Nicholl J. (2008). The quality of mixed methods studies in health services research. J. Health Serv. Res. Policy.

[27] Braun V., Clarke V. (2021). One size fits all? What counts as quality practice in (reflexive) thematic analysis?. Qual. Res. Psychol..

[28] Blanco M., Prunuske J., DiCorcia M., Learman L.A., Mutcheson B., Huang G.C. (2022). The DoCTRINE Guidelines: Defined Criteria To Report INnovations in Education. Acad. Med..

[29] Downing S.M. (2003). Validity: On meaningful interpretation of assessment data. Med. Educ..

[30] Cook D.A., Beckman T.J. (2006). Current concepts in validity and reliability for psychometric instruments: Theory and application. Am. J. Med..

[31] Dorrestein L., Ritter C., De Mol Z., Wichtel M., Cary J., Vengrin C., Artemiou E., Adams C.L., Ganshorn H., Coe J.B. (2025). Validity evidence for communication skills assessment in health professions education: A scoping review. BMJ Open.

[32] Zerbini G., Schneider P., Reicherts M., Roob N., Jung-Can K., Kunz M., Reicherts P. (2025). A novel multi-measure approach to study medical students’ communication performance and predictors of their communication quality—A cross-sectional study. BMC Med. Educ..

