# Supplementary material for: The Micro-Expression Decoder: A Preliminary Mixed-Methods Evaluation of Training in Subtle Facial and Non-Verbal Cue Recognition in Final-Year Medical Students
Source: Clin Pract. 2026 Sep 14;16(9):170. doi: 10.3390/clinpract16090170 (PMC13605176; doi:10.3390/clinpract16090170)

Supplementary materials

Supplementary Table S1: Operational cue taxonomy and construct mapping.

Construct	Operational description	Examples in MED	Role in interpretation
Strict micro-expression	Brief involuntary facial movement occurring over a fraction of a second	Lip compression; widened eyes; facial tension; raised eyebrows	Facial behaviour was treated as a possible prompt, never as proof of emotion
Broader facial/non-verbal cue	Observable interactional behaviour not technically a micro-expression	Uncertain gaze; hesitant nodding; hesitant pause	Explicitly labelled as broader interactional cues

Supplementary Table S2: Outcome map and analysis plan.

Status	Outcome	Source/scoring	Analysis	Reported in
Primary	Confidence recognising subtle facial cues	Student item Q1; 1–5	Continuous paired change; descriptive dichotomy 4–5 vs 1–3	Table 3; Figure 2
Primary	Confidence interpreting possible emotional significance	Student item Q2; 1–5	Continuous paired change; descriptive dichotomy 4–5 vs 1–3	Table 3; Figure 2
Secondary	SP-rated attentiveness	SP1; 1–5 single item	Paired t-test; Wilcoxon; d_z; ceiling	Table 3; Figure 2
Secondary	SP-rated satisfaction	SP8; 1–5 single item	Paired t-test; Wilcoxon; d_z; ceiling	Table 3; Figure 2
Secondary	Anxiety recognition	At least 2/3 anxiety clips correct; category + response both acceptable	Exact McNemar; transitions	Table 4; Figure 3
Secondary	Confusion recognition	At least 2/3 confusion clips correct; category + response both acceptable	Exact McNemar; transitions	Table 4; Figure 3
Secondary	Confidence using non-verbal communication	Student Q6; 4–5 meets criterion	Exact McNemar; transitions	Table 4; Figure 3
Secondary	Importance for rapport-building	Student Q7; 4–5 meets criterion	Exact McNemar; transitions	Table 4; Figure 3
Exploratory	Awareness of influence on interactions	Student Q3; 1–5	Paired t-test; Wilcoxon; maximum-score proportion	Table S3
Exploratory	Self-rated ability to identify anxiety-related cues	Student Q4; 1–5	Paired t-test; Wilcoxon; maximum-score proportion	Table S3
Exploratory	Self-rated ability to identify confusion-related cues	Student Q5; 1–5	Paired t-test; Wilcoxon; maximum-score proportion	Table S3
Exploratory	Active observation of facial expressions	Student Q8; 1–5	Paired t-test; Wilcoxon; maximum-score proportion	Table S3
Exploratory	Ability to adapt communication	Student Q9; 1–5	Paired t-test; Wilcoxon; maximum-score proportion	Table S3
Exploratory	Uncertainty recognition	Single uncertainty clip correct; category + response both acceptable	Exact McNemar; transitions	Table 4; Figure 3
Exploratory	Remaining interpretable SP items	SP2-SP5 and SP7; 1-5 single items	Exploratory paired t-test; Wilcoxon signed-rank test; maximum-score proportion	Supplementary Table S4
Descriptive only	SP6, original administered wording	1-5 single item	Initial and final descriptive values only; no paired inferential statistic or effect estimate	Supplementary Table S4 and Instrument S12

Supplementary Table S3: Results for all nine student questionnaire items.

Item	Label	Initial: mean ± SD; median [IQR]	Immediate final: mean ± SD; median [IQR]	Mean change (95% CI)	Paired t-test	Wilcoxon	Maximum initial/final
Q1	Confidence recognising subtle facial cues	2.70 ± 0.91; 3.0 [2–3]	4.30 ± 0.62; 4.0 [4–5]	1.60 (1.39–1.81)	t(59)=15.36; p<0.001	W=0.0; p<0.001	0.0% / 38.3%
Q2	Confidence interpreting possible emotional significance	3.00 ± 0.80; 3.0 [2–3]	4.50 ± 0.50; 4.5 [4–5]	1.50 (1.31–1.69)	t(59)=15.54; p<0.001	W=0.0; p<0.001	5.0% / 50.0%
Q3	Awareness of influence on interactions	3.12 ± 0.78; 3.0 [3–4]	4.40 ± 0.81; 5.0 [4–5]	1.28 (1.11–1.46)	t(59)=14.92; p<0.001	W=0.0; p<0.001	0.0% / 58.3%
Q4	Self-rated ability to identify anxiety-related cues	2.93 ± 0.76; 3.0 [2–3]	4.42 ± 0.79; 5.0 [4–5]	1.48 (1.30–1.66)	t(59)=16.39; p<0.001	W=0.0; p<0.001	1.7% / 58.3%
Q5	Self-rated ability to identify confusion-related cues	3.03 ± 0.80; 3.0 [3–4]	4.53 ± 0.79; 5.0 [4–5]	1.50 (1.34–1.66)	t(59)=18.61; p<0.001	W=0.0; p<0.001	0.0% / 68.3%
Q6	Confidence using non-verbal communication	2.93 ± 1.21; 3.0 [2–4]	4.08 ± 0.81; 4.0 [4–5]	1.15 (0.84–1.46)	t(59)=7.39; p<0.001	W=80.5; p<0.001	13.3% / 30.0%
Q7	Importance for rapport-building	3.52 ± 1.30; 3.5 [3–5]	4.40 ± 0.62; 4.0 [4–5]	0.88 (0.55–1.22)	t(59)=5.25; p<0.001	W=121.5; p<0.001	31.7% / 46.7%
Q8	Active observation of facial expressions	2.93 ± 0.82; 3.0 [2–3]	4.40 ± 0.67; 4.5 [4–5]	1.47 (1.30–1.63)	t(59)=17.48; p<0.001	W=0.0; p<0.001	3.3% / 50.0%
Q9	Ability to adapt communication	3.25 ± 0.77; 3.0 [3–4]	4.57 ± 0.77; 5.0 [4–5]	1.32 (1.14–1.50)	t(59)=14.55; p<0.001	W=0.0; p<0.001	5.0% / 71.7%

Supplementary Table S4: Results for all eight standardised-patient items.

Item	Label	Initial: mean ± SD; median [IQR]	Immediate final: mean ± SD; median [IQR]	Mean change (95% CI)	Paired t-test	Wilcoxon	Maximum initial/final
SP1	Attentiveness to non-verbal cues	3.20 ± 0.71; 3.0 [3–4]	4.60 ± 0.49; 5.0 [4–5]	1.40 (1.22–1.58)	t(59)=15.63; p<0.001	W=0.0; p<0.001	3.3% / 60.0%
SP2	Recognition of anxiety cues	2.98 ± 0.81; 3.0 [2–4]	4.42 ± 0.72; 5.0 [4–5]	1.43 (1.27–1.59)	t(59)=17.89; p<0.001	W=0.0; p<0.001	1.7% / 55.0%
SP3	Recognition of confusion/uncertainty cues	3.12 ± 0.85; 3.0 [3–4]	4.32 ± 0.75; 4.0 [4–5]	1.20 (0.99–1.41)	t(59)=11.64; p<0.001	W=15.5; p<0.001	3.3% / 46.7%
SP4	Adaptation of communication	2.97 ± 0.71; 3.0 [2–3]	4.48 ± 0.77; 5.0 [4–5]	1.52 (1.34–1.70)	t(59)=16.76; p<0.001	W=0.0; p<0.001	0.0% / 61.7%
SP5	Pausing/clarifying when uncertain	3.18 ± 0.75; 3.0 [3–4]	4.62 ± 0.69; 5.0 [4–5]	1.43 (1.26–1.61)	t(59)=16.49; p<0.001	W=16.5; p<0.001	0.0% / 71.7%
SP6	Original comparative wording; descriptive only	3.25 ± 0.68; 3.0 [3–4]	4.60 ± 0.56; 5.0 [4–5]	Not calculated	Not tested	Not tested	3.3% / 63.3%
SP7	Feeling understood and respected	3.45 ± 0.67; 3.0 [3–4]	4.68 ± 0.60; 5.0 [4–5]	1.23 (1.07–1.39)	t(59)=15.39; p<0.001	W=0.0; p<0.001	5.0% / 73.3%
SP8	Overall satisfaction	3.50 ± 0.79; 4.0 [3–4]	4.80 ± 0.40; 5.0 [5–5]	1.30 (1.13–1.47)	t(59)=15.00; p<0.001	W=0.0; p<0.001	8.3% / 80.0%

Footnote: SP6 was administered unchanged at both encounters. Because the wording "improved the quality of the interaction" was comparative and had no valid reference point at the first encounter, no paired inferential statistic or effect estimate is reported. The item is retained only to document the instrument that participants received.

Supplementary Table S5: Recognition-task clip map and prespecified answer guide. Table S5 describes the 14 recognition-assessment clips and does not describe the teaching-clip set.

Clip code	Source/provenance record	Observable cue	Target category	Acceptable clinical response	Domain	Parallel-form documentation
Pre A1	Licensed educational acted clip; original provider/item metadata not retained	Lip compression	Anxiety	Acknowledge possible worry and ask an open question	Anxiety	Matched with Post A1 on cue type, duration and intended intensity
Pre A2	Purpose-recorded acted clip; MED study archive	Widened eyes	Anxiety	Pause and check how the patient is feeling	Anxiety	Matched with Post A2 on cue type, duration and intended intensity
Pre A3	Licensed educational acted clip; original provider/item metadata not retained	Facial tension	Anxiety	Use tentative language and invite elaboration	Anxiety	Matched with Post A3 on cue type, duration and intended intensity
Pre C1	Purpose-recorded acted clip; MED study archive	Raised eyebrows	Confusion	Rephrase and check understanding	Confusion	Matched with Post C1 on cue type, duration and intended intensity
Pre C2	Licensed educational acted clip; original provider/item metadata not retained	Hesitant nodding	Confusion	Ask the patient to explain in their own words	Confusion	Matched with Post C2 on cue type, duration and intended intensity
Pre C3	Purpose-recorded acted clip; MED study archive	Uncertain gaze	Confusion	Pause and clarify the explanation	Confusion	Matched with Post C3 on cue type, duration and intended intensity
Pre U1	Licensed educational acted clip; original provider/item metadata not retained	Hesitant pause	Uncertainty	Acknowledge the pause and ask what is concerning them	Uncertainty	Matched with Post U1 on cue type, duration and intended intensity
Post A1	Purpose-recorded acted clip; MED study archive	Lip compression	Anxiety	Acknowledge possible worry and ask an open question	Anxiety	Parallel to Pre A1; matched on cue type, duration and intended intensity
Post A2	Licensed educational acted clip; original provider/item metadata not retained	Widened eyes	Anxiety	Pause and check how the patient is feeling	Anxiety	Parallel to Pre A2; matched on cue type, duration and intended intensity
Post A3	Purpose-recorded acted clip; MED study archive	Facial tension	Anxiety	Use tentative language and invite elaboration	Anxiety	Parallel to Pre A3; matched on cue type, duration and intended intensity
Post C1	Licensed educational acted clip; original provider/item metadata not retained	Raised eyebrows	Confusion	Rephrase and check understanding	Confusion	Parallel to Pre C1; matched on cue type, duration and intended intensity
Post C2	Purpose-recorded acted clip; MED study archive	Hesitant nodding	Confusion	Ask the patient to explain in their own words	Confusion	Parallel to Pre C2; matched on cue type, duration and intended intensity
Post C3	Licensed educational acted clip; original provider/item metadata not retained	Uncertain gaze	Confusion	Pause and clarify the explanation	Confusion	Parallel to Pre C3; matched on cue type, duration and intended intensity
Post U1	Purpose-recorded acted clip; MED study archive	Hesitant pause	Uncertainty	Acknowledge the pause and ask what is concerning them	Uncertainty	Parallel to Pre U1; matched on cue type, duration and intended intensity

Video-stimulus provenance and scoring. The recognition-assessment set comprised seven licensed educational acted clips and seven purpose-recorded acted clips. These clips were separate from the 14 teaching clips, which comprised eight licensed educational clips and six purpose-recorded clips; no recognition-assessment clip was shown or discussed during teaching. The assessment clips are identified using the study codes retained in the MED documentation. Purpose-recorded material formed part of the MED study archive. For the licensed educational clips, the original commercial provider, product-level identifier and acquisition metadata were not retained in the study archive and have therefore not been reconstructed retrospectively. The licensed video files cannot be redistributed because of copyright restrictions. Initial and final assessment forms used parallel rather than identical clips and were matched prospectively by target category, observable cue, duration and intended intensity. A clip was scored as correct only when both the selected category and clinical response matched the prespecified guide. Anxiety and confusion outcomes required at least two of the three relevant clips to be correct; the single uncertainty clip was treated as an exploratory outcome..

Supplementary Table S6: Underlying categorical transition counts.

Outcome	Initial not met / immediate final not met	Initial not met / immediate final met	Initial met / immediate final not met	Initial met / immediate final met	Exact McNemar p
Non-verbal confidence	9	33	0	18	<0.001
Rapport importance	4	26	0	30	<0.001
Anxiety recognition	5	30	0	25	<0.001
Confusion recognition	7	24	1	28	<0.001
Uncertainty recognition	11	25	6	18	<0.001

Supplementary Table S7: Fidelity by session.

Session	Applicable items	Items delivered	Fidelity	Observer
G01	17	15	88.2%	OBS1
G02	17	16	94.1%	OBS1
G03	17	16	94.1%	OBS2
G04	17	15	88.2%	OBS1
G05	17	17	100.0%	OBS2
G06	17	16	94.1%	OBS2
G07	17	16	94.1%	OBS1
G08	17	17	100.0%	OBS2
G09	17	15	88.2%	OBS1
G10	17	16	94.1%	OBS2

Mean fidelity was 93.5% (rounded to 94% in the manuscript), with a range of 88.2%–100.0%.

Supplementary Table S8: Continuous-outcome clustering sensitivity analyses.

Grouping variable	Outcome	Range of stratum mean changes	F	p	η²
School	Recognition confidence	1.50–1.68	0.262	0.771	0.009
School	Interpretation confidence	1.28–1.64	1.214	0.305	0.041
School	SP attentiveness	1.22–1.50	0.867	0.425	0.030
School	SP satisfaction	1.23–1.40	0.353	0.704	0.012
Session	Recognition confidence	1.33–2.00	0.370	0.944	0.063
Session	Interpretation confidence	1.17–2.00	0.624	0.771	0.101
Session	SP attentiveness	1.00–1.67	0.592	0.798	0.096
Session	SP satisfaction	0.83–1.83	1.061	0.407	0.160
Final-encounter SP	Recognition confidence	1.33–1.89	0.612	0.691	0.054
Final-encounter SP	Interpretation confidence	1.00–1.85	1.968	0.098	0.154
Final-encounter SP	SP attentiveness	1.00–1.70	1.726	0.144	0.138
Final-encounter SP	SP satisfaction	1.11–1.50	0.474	0.794	0.042

Assessor-related analyses were stratified by the final-encounter standardised patient. Because a different standardised patient rated the first encounter, these analyses do not capture all possible first/final assessor-pair effects. These one-way analyses are exploratory. The small numbers of schools, sessions and assessors limit precision and do not exclude clustering.

Supplementary Table S9: Binary-improvement clustering sensitivity analyses.

Grouping variable	Outcome	χ²	p
School	Anxiety recognition	1.527	0.466
School	Confusion recognition	0.606	0.739
School	Uncertainty recognition	0.154	0.926
Session	Anxiety recognition	5.333	0.804
Session	Confusion recognition	7.222	0.614
Session	Uncertainty recognition	5.829	0.757
Final-encounter SP	Anxiety recognition	6.677	0.246
Final-encounter SP	Confusion recognition	7.094	0.214
Final-encounter SP	Uncertainty recognition	5.885	0.318

Assessor-related analyses were stratified by the final-encounter standardised patient. Because a different standardised patient rated the first encounter, these analyses do not capture all possible first/final assessor-pair effects.

Supplementary Table S10: Qualitative coding framework and illustrative positive and discrepant extracts.

Type	Theme/code	Verbatim extract	Anonymous source
Core theme	Recognition of possible anxiety	I noticed the patient looked anxious when they pressed their lips together, which helped me address their concerns.	Student 14, reflective journal
Core theme	Clarification in response to confusion	The patient's raised eyebrows made me realise they did not understand my explanation, so I rephrased it.	Student 27, reflective journal
Core theme	Checking rather than assuming	It reminded me to check what the patient was feeling instead of deciding for them.	Student 41, post-training open response
Core theme	Enhanced engagement and presence	The student noticed my nervousness and paused to ask how I was feeling. It made the interaction feel more personal.	SP 3, post-training comment
Core theme	Reflective growth	I used to miss these subtle signs completely. Now I actively look for them.	Student 08, reflective journal
Core theme	Intended transfer to future practice	I plan to look for these cues during sensitive conversations.	Student 52, reflective journal
Negative/discrepant case	Difficulty distinguishing cues	At first I could not tell whether the patient was anxious or simply confused, so I had to ask rather than rely on the expression.	Student 19, reflective journal
Negative/discrepant case	Risk of over-interpretation	I realised I had read too much into one pause. The safer approach was to acknowledge it and check what it meant.	Student 33, post-training open response
Negative/discrepant case	Competing attentional demands	Concentrating on facial cues sometimes distracted me from the history, particularly when I was trying to structure the consultation.	Student 47, reflective journal
Negative/discrepant case	Cultural variation	The same expression may not mean the same thing for every patient or culture, so the cue cannot be interpreted on its own.	Student 05, reflective journal
Negative/discrepant case	Limited perceived change	The session made me more aware, but I am not yet sure that I would recognise these cues reliably in a busy clinic.	Student 58, post-training open response
Negative/discrepant case	Missed cue	The student responded well overall but did not notice the first hesitant pause until I repeated it.	SP 6, post-training comment

Frequencies represent the number of distinct source records containing a theme. Approximately 20% of each source type was independently double-coded: 12/56 journals (21.4%), 12/60 post-training open responses (20.0%), 12/60 standardised-patient comments (20.0%) and 2/10 group-debrief records (20.0%). The sample covered all ten groups and all three schools.

Supplementary instrument S11: Student evaluation form

The wording below is reproduced verbatim as administered. The original instrument retained the term "micro-expressions" in several items.

Response scale: 1 = strongly disagree; 2 = disagree; 3 = neutral; 4 = agree; 5 = strongly agree.

Item	Statement	1	2	3	4	5
1	I feel confident in recognising subtle facial expressions indicating a patient's emotions.	□	□	□	□	□
2	I feel confident in interpreting the emotional significance of patients' facial expressions.	□	□	□	□	□
3	I am aware of how micro-expressions can influence patient–clinician interactions.	□	□	□	□	□
4	I can identify anxiety-related facial cues during clinical interactions.	□	□	□	□	□
5	I can identify confusion-related facial cues during clinical interactions.	□	□	□	□	□
6	I feel confident using non-verbal communication skills during patient interactions.	□	□	□	□	□
7	I believe recognising micro-expressions is important for building rapport with patients.	□	□	□	□	□
8	I actively observe patients' facial expressions during clinical interactions.	□	□	□	□	□
9	I feel able to adapt my communication when patients appear anxious or uncertain.	□	□	□	□	□

Pre-training open questions

1. What do you currently know about micro-expressions and their role in clinical practice?
2. What do you hope to gain from this training programme?
3. What challenges do you anticipate in identifying and interpreting micro-expressions?
4. How confident are you in responding to patients' unspoken emotional cues?

Post-training open questions

1. What was the most valuable skill you learned from this training?
2. How has this training changed the way you approach patient interactions?
3. What strategies will you use to integrate micro-expression awareness into your future clinical practice?
4. Describe how recognising subtle emotional cues affected your communication during role-play encounters.

Supplementary instrument S12: Standardised-patient feedback form

Response scale: 1 = strongly disagree; 2 = disagree; 3 = neutral; 4 = agree; 5 = strongly agree.

Item	Statement	1	2	3	4	5
1	The student demonstrated attentiveness to my non-verbal cues during the interaction.	□	□	□	□	□
2	The student accurately recognised my subtle expressions of anxiety.	□	□	□	□	□
3	The student accurately recognised my subtle expressions of confusion or uncertainty.	□	□	□	□	□
4	The student adapted their communication based on my non-verbal cues.	□	□	□	□	□
5	The student paused or clarified information when I appeared uncertain.	□	□	□	□	□
6	The student's responsiveness to my facial expressions improved the quality of the interaction.	□	□	□	□	□
7	The student made me feel understood and respected during the interaction.	□	□	□	□	□
8	Overall, I was satisfied with the interaction.	□	□	□	□	□

Item 6 is reproduced exactly as administered; its comparative wording was unsuitable at the first encounter and the item was excluded from paired inference. Recommended wording for future administration: "The student's response to my facial expressions supported the quality of the interaction."

Open-ended questions

1. What did the student do particularly well during this interaction?
2. Were there any non-verbal cues the student missed or misinterpreted? Please explain.
3. How did the student's attentiveness to your non-verbal cues affect your overall experience?
4. Did the student demonstrate empathy or clarification when you appeared uncertain or anxious?

Supplementary instrument S13: Reflective journal prompts

1. Describe a specific moment during the role-playing exercises when you successfully identified a patient's micro-expression. How did this influence your interaction?
2. Reflect on a situation where you missed or misinterpreted a micro-expression. What did you learn from this experience?
3. Describe how recognising signs of anxiety or uncertainty affected the way you communicated with the patient.
4. Describe a moment when you changed or clarified your explanation because of a patient's facial expression or body language.
5. How has this training changed your approach to observing and interpreting non-verbal communication in clinical practice?
6. Did the training alter your understanding of empathy, attentiveness or rapport-building? Please explain.
7. What challenges do you anticipate in applying these skills in real clinical settings, and how might you address them?
8. Suggest strategies for integrating cue awareness into routine clinical interactions to improve communication.
9. In what types of consultations do you think these skills will be most useful in future practice?
10. Reflect on how this training may influence your future communication style as a clinician.

Supplementary note S14: Administrative and analytic details

Field	Verified detail
Programme duration	270 minutes total: 255 structured activity + 15-minute break
Timetable	Consent and initial student measures, 20 minutes; conceptual workshop, 35 minutes; video observation and cue labelling, 40 minutes; first simulated encounter and rating, 15 minutes; structured teaching and paired practice, 55 minutes; scheduled break, 15 minutes; final simulated encounter, rating and feedback, 45 minutes; final recognition task and survey, 25 minutes; debrief and reflective writing, 20 minutes.
Feedback timing	SP ratings were completed before feedback at both simulated encounters. Brief formative feedback followed the first encounter; full structured SP and educator feedback followed the final encounter.
Debrief	20 minutes including reflective-journal writing; description–analysis–application sequence
Verified session months	October and December 2023; March, June, September and November 2024; February, May, September and November 2025
Data retention	10 years after publication, then secure destruction
Power software	G*Power 3.1.9.7; two-sided paired-samples t-test; dz=0.50; α=0.05; power=0.80; minimum N=34. No separate pre–post correlation was entered because the calculation was parameterised directly using dz. The effect was a conventional moderate planning assumption rather than an estimate from prior MED data
Qualitative software	NVivo 15
Qualitative analysts	Analyst 1: clinician-medical educator involved in MED development; Analyst 2: independent qualitative health-researcher not involved in delivery
Double coding	12/56 journals; 12/60 post-training open responses; 12/60 SP comments; 2/10 group debrief records; included all 10 groups and all three schools
Educators	Four unique educators: two GP/medical educators, one simulation fellow, one communication-skills psychologist; two educators per session, not always the same pair
Cognitive pilot	8 students, excluded from final study
Intervention pilot	6 learners, excluded from final study
SP rehearsal	90-minute rehearsal in addition to the two-hour calibration session
Video sources	Teaching clips: 14 total—8 licensed and 6 purpose-recorded. Recognition-assessment clips: 14 total—7 licensed and 7 purpose-recorded. The sets were separate and no assessment clip was used during teaching.
Fidelity observers	Two trained independent observers shared the ten sessions using the same 17-item checklist
Potentially eligible population	Not recorded; administrators supplied invited counts only
Governance	The HRA decision tool was completed prospectively on 10 August 2023, before recruitment and before the first study session. It classified the project as research and indicated that NHS REC review was not required for sites in England.

Supplementary Instrument S15: Recognition-task response forms

S15.1 Administration instructions

Students complete one initial form and one immediate-final form. Each form contains seven short acted clips: three anxiety-targeted clips, three confusion-targeted clips and one uncertainty-targeted clip. Initial and final clips are parallel rather than identical.

- Each clip is shown once in the prespecified order; no teaching, discussion or corrective feedback occurs during the task.
- For each clip, select one cue category and one clinical-response code. The cue should be treated as a prompt for enquiry, not as proof of a patient's internal state.
- Select only one category and one response code. A blank answer or more than one selection in either part is scored as incorrect.
- The initial form is completed before teaching. The immediate-final form is completed after the instructional and simulation components and before the debrief.

S15.2 Cue-category options

Code	Category	Participant-facing description
**A**	Anxiety	The cue may indicate possible worry, apprehension or concern.
**C**	Confusion	The cue may indicate possible misunderstanding or difficulty following the explanation.
**U**	Uncertainty	The cue may indicate hesitation or uncertainty without enough information to classify it as anxiety or confusion.
**D**	Discomfort	The cue may indicate possible physical or interpersonal discomfort.
**X**	Unsure	There is insufficient information to select another category.

S15.3 Clinical-response option bank

Code	Clinical response
**R1**	Acknowledge possible worry and ask an open question.
**R2**	Pause and check how the patient is feeling.
**R3**	Use tentative language and invite the patient to elaborate.
**R4**	Rephrase the information and check understanding.
**R5**	Ask the patient to explain the information or plan in their own words.
**R6**	Pause and clarify the explanation.
**R7**	Acknowledge the pause and ask what is concerning the patient.
**R8**	State the presumed emotion as a fact, without checking.
**R9**	Continue the consultation without responding to the cue.
**R10**	Offer immediate reassurance without first checking what the cue means.
**R11**	Change the subject or move directly to the next task.

S15.4 Initial recognition-task response form

Participant identifier: ____________________ Session: __________ Date: __________ Form: Initial

Clip order	Administrative clip code*	Select one cue category	Response code	Assessor use only
1	Pre A1	[ ] A [ ] C [ ] U [ ] D [ ] X	R____	
2	Pre A2	[ ] A [ ] C [ ] U [ ] D [ ] X	R____	
3	Pre A3	[ ] A [ ] C [ ] U [ ] D [ ] X	R____	
4	Pre C1	[ ] A [ ] C [ ] U [ ] D [ ] X	R____	
5	Pre C2	[ ] A [ ] C [ ] U [ ] D [ ] X	R____	
6	Pre C3	[ ] A [ ] C [ ] U [ ] D [ ] X	R____	
7	Pre U1	[ ] A [ ] C [ ] U [ ] D [ ] X	R____	

**Administrative clip codes are shown here for documentation and were not displayed on the participant-facing form.*

S15.5 Immediate-final recognition-task response form

Participant identifier: ____________________ Session: __________ Date: __________ Form: Immediate final

Clip order	Administrative clip code*	Select one cue category	Response code	Assessor use only
1	Post A1	[ ] A [ ] C [ ] U [ ] D [ ] X	R____	
2	Post A2	[ ] A [ ] C [ ] U [ ] D [ ] X	R____	
3	Post A3	[ ] A [ ] C [ ] U [ ] D [ ] X	R____	
4	Post C1	[ ] A [ ] C [ ] U [ ] D [ ] X	R____	
5	Post C2	[ ] A [ ] C [ ] U [ ] D [ ] X	R____	
6	Post C3	[ ] A [ ] C [ ] U [ ] D [ ] X	R____	
7	Post U1	[ ] A [ ] C [ ] U [ ] D [ ] X	R____	

**Administrative clip codes are shown here for documentation and were not displayed on the participant-facing form.*

S15.6 Prespecified scoring key

Clip pair	Observable cue	Correct category	Correct response	Acceptable response description
Pre A1 / Post A1	Lip compression	Anxiety (A)	R1	Acknowledge possible worry and ask an open question.
Pre A2 / Post A2	Widened eyes	Anxiety (A)	R2	Pause and check how the patient is feeling.

Clip pair	Observable cue	Correct category	Correct response	Acceptable response description
Pre A3 / Post A3	Facial tension	Anxiety (A)	R3	Use tentative language and invite elaboration.
Pre C1 / Post C1	Raised eyebrows	Confusion (C)	R4	Rephrase and check understanding.
Pre C2 / Post C2	Hesitant nodding	Confusion (C)	R5	Ask the patient to explain in their own words.
Pre C3 / Post C3	Uncertain gaze	Confusion (C)	R6	Pause and clarify the explanation.
Pre U1 / Post U1	Hesitant pause	Uncertainty (U)	R7	Acknowledge the pause and ask what is concerning the patient.

S15.7 Domain scoring rules

- Item-level score: 1 only when both the cue category and clinical-response code match the prespecified key; otherwise 0. No partial credit is assigned.
  - Total raw score: the seven item-level scores are summed, giving a range of 0-7 for each form.
  - Anxiety domain score: 0-3. Confusion domain score: 0-3. Uncertainty item: 0-1 and exploratory.
  - The criterion outcomes remain at least two of three correct for anxiety and confusion; the raw total is descriptive and was not a prespecified primary or secondary outcome.
- Anxiety recognition: criterion met when at least two of the three anxiety items are correct.
- Confusion recognition: criterion met when at least two of the three confusion items are correct.
- Uncertainty recognition: the single uncertainty item is reported as an exploratory binary outcome.
- Missing or multiple selections were scored as incorrect. Alternative responses were accepted only when they had been documented in the prespecified archived scoring guide.

Supplementary Table S16: Simulated-encounter scenario and cue map

S16.1 Scenario-level matching framework

Dimension	First encounter (pre-practice)	Final encounter	Matching rule
**Encounter duration**	8 minutes	8 minutes	Identical
**Clinical task**	Explain a new diagnosis and initial treatment plan	Explain a new diagnosis and initial treatment plan	Matched communication demand
**Clinical content**	New hypertension diagnosis and first-line medication	New asthma diagnosis and preventer inhaler	Different content to reduce simple recall
**Key information units**	Diagnosis, treatment, monitoring/safety-netting	Diagnosis, treatment, monitoring/safety-netting	Three matched information units
**Planned cue episodes**	Three	Three	One anxiety, one confusion and one uncertainty episode
**Target cue intensity**	Subtle-to-moderate (2/3)	Subtle-to-moderate (2/3)	Matched during SP rehearsal
**SP allocation**	First-encounter SP	Different final-encounter SP	Reduces interpersonal familiarity
**Rating and feedback**	SP rating before brief formative feedback	SP rating before structured feedback	Same rating-before-feedback rule

S16.2 Illustrative cue map

Encounter	Scenario synopsis	Scripted trigger	Planned observable cue	Target domain	Timing/intensity	Standardised SP line if checked	Expected communication response
**First encounter**	New hypertension diagnosis and first-line medication	After the student explains that repeated blood-pressure readings support a diagnosis of hypertension	Lip compression with brief facial tension	Possible anxiety	Subtle-to-moderate; hold for 2–3 seconds	"I did not expect this. I am worried it means something serious."	Tentatively acknowledge possible worry and invite an open response
**First encounter**	New hypertension diagnosis and first-line medication	After the student explains daily dosing and monitoring	Raised eyebrows followed by hesitant nodding	Possible confusion	Subtle-to-moderate; one cue sequence	"I am not sure whether I take it every day or only when the reading is high."	Rephrase, check understanding, and use teach-back
**First encounter**	New hypertension diagnosis and first-line medication	At the first natural closing point	Hesitant pause with uncertain gaze	Uncertainty	Subtle; one pause of approximately 2 seconds	"I am worried about side effects, but I did not want to hold things up."	Acknowledge the pause and ask what remains concerning
**Final encounter**	New asthma diagnosis and preventer inhaler	After the student explains the diagnosis and treatment rationale	Widened eyes with brief facial tension	Possible anxiety	Subtle-to-moderate; hold for 2–3 seconds	"I am worried that I might suddenly stop breathing."	Pause, check how the patient feels, and invite elaboration
**Final encounter**	New asthma diagnosis and preventer inhaler	After the student distinguishes the preventer and reliever inhalers	Hesitant nodding with uncertain gaze	Possible confusion	Subtle-to-moderate; one cue sequence	"I am not clear which inhaler is for every day and which one is for symptoms."	Rephrase and ask the patient to explain the plan in their own words
**Final encounter**	New asthma diagnosis and preventer inhaler	Before safety-netting and closure	Hesitant pause	Uncertainty	Subtle; one pause of approximately 2 seconds	"I am not sure what should make me seek urgent help."	Acknowledge the pause, clarify, and complete safety-netting

S16.3 Standardisation rules for SP delivery

- The SP presents the cue only after the specified verbal trigger. The cue is not exaggerated to ensure recognition.
- If the student notices and checks the cue, the SP gives the scripted line, answers the student's follow-up naturally within the scenario, and does not repeat the cue.
- If the cue is missed, the SP may repeat it once at the next natural conversational opportunity, using the same intensity. A second repetition is not permitted.
- The SP does not coach, praise, correct or explain the cue during the assessed encounter. The rating form is completed before any feedback.
- Cue intensity and the acceptability of the scripted lines are calibrated during the two-hour SP training session and 90-minute rehearsal.

Supplementary Instrument S17: Seventeen-item fidelity checklist

Session code: __________ Date: __________ School: __________ Observer: [ ] OBS1 [ ] OBS2 Educators: ____________________ Group size: ____

Observer instructions

- Complete one checklist contemporaneously for each session. The observer should not be one of the two educators delivering that session.
- Score “1” only when the item is observed in the prescribed form. Score “0” when absent, incomplete or materially altered, and record the deviation.
- For the study as reported, all 17 items were applicable in every session. Do not use “not applicable” unless a documented protocol amendment justifies it.
- Session fidelity = number of items delivered / 17 × 100. Thus, 15/17 = 88.2%, 16/17 = 94.1%, and 17/17 = 100.0%.

No.	Domain	Fidelity item	Yes (1)	No (0)	Observed deviation/evidence
1	Assessment timing	Written consent and the initial student survey and recognition task were completed before any teaching.	[ ]	[ ]	
2	Programme timing	The prescribed 270-minute sequence was followed, including 255 minutes of structured activity and a 15-minute scheduled break.	[ ]	[ ]	
3	Construct definition	The workshop distinguished strict micro-expressions from broader facial and interactional cues.	[ ]	[ ]	
4	Ethical framing	Facial and interactional cues were taught as possible prompts for enquiry, not as diagnostic proof of emotion.	[ ]	[ ]	
5	MED pathway	The notice–interpret–check–adapt pathway was explicitly taught.	[ ]	[ ]	
6	Teaching clips	All 14 teaching clips were shown in the prescribed sequence.	[ ]	[ ]	
7	Assessment separation	No recognition-assessment clip was shown or discussed during teaching.	[ ]	[ ]	
8	Cue labelling	Students completed individual cue labelling before facilitated group discussion.	[ ]	[ ]	
9	Context and ambiguity	The discussion addressed ambiguity, alternative meanings, cultural variation and the risk of over-interpretation.	[ ]	[ ]	
10	First encounter	The first eight-minute simulated encounter was delivered using the prescribed parallel scenario and planned cues.	[ ]	[ ]	
11	First rating	The SP completed the rating form before any formative feedback was given.	[ ]	[ ]	
12	First feedback	Brief formative feedback followed the first encounter and used the common feedback rubric.	[ ]	[ ]	
13	Structured practice	The 55-minute structured teaching and paired-practice component covered noticing, tentative interpretation, direct checking and communication adaptation.	[ ]	[ ]	
14	Final encounter	The final eight-minute parallel encounter was delivered by a different SP using the prescribed matched cues.	[ ]	[ ]	

No.	Domain	Fidelity item	Yes (1)	No (0)	Observed deviation/evidence
15	Final rating/feedback	The final SP rating was completed before structured SP and educator feedback.	[ ]	[ ]	
16	Final measures	The parallel-form recognition task and immediate-final student survey were completed before the debrief.	[ ]	[ ]	
17	Debrief/reflection	The description–analysis–application debrief and reflective-journal activity were completed using the standard template.	[ ]	[ ]	

Session score

Items delivered: ____ / 17 Fidelity: ______ % Material deviation requiring review: [ ] No [ ] Yes

Observer notes and corrective action for subsequent sessions:

____

____

____

Supplementary Instrument S18: Feedback rubric and debrief structure

S18.1 Feedback principles

- The SP completes the quantitative rating form before any feedback or discussion.
- Feedback describes observable behaviour and its effect within the simulation; it does not claim access to the SP's "true" emotion or the student's clinical competence.
- Cue recognition, tentative interpretation, direct checking and communication adaptation are discussed as separate steps.
- Feedback should include one specific strength, one calibration point and one actionable feed-forward step.
- The learner is reminded that the same cue can have several meanings and that checking is safer than confident inference.

S18.2 Standardised-patient formative feedback rubric

Domain	Effective behaviour	Calibration concern	Illustrative feedback stem
**Attentiveness/noticing**	Student notices a change in expression, gaze, nodding or pause without abandoning the consultation task.	Cue is missed, noticed only after repetition, or monitored so intensely that history-taking is disrupted.	"When I paused and looked uncertain, you slowed down and gave me space to respond."
**Tentative interpretation**	Student uses qualified language and allows several possible meanings.	Student labels the emotion as certain or makes a deterministic statement.	"You checked what the cue might mean rather than telling me what I was feeling."
**Checking/clarification**	Student asks an open or teach-back question that tests understanding or concern.	Student relies on a leading yes/no question, generic reassurance or assumption.	"A more useful check would have been to ask me to explain the plan in my own words."
**Communication adaptation**	Student rephrases, pauses, changes pace, clarifies or safety-nets in response to the checked meaning.	Student notices the cue but continues with the same explanation and pace.	"After I said I was confused, the shorter explanation helped me follow the plan."
**Respect and presence**	Student remains non-judgemental, maintains rapport and gives the SP time to answer.	Student appears impatient, corrective or dismissive.	"I felt able to raise the concern because you acknowledged the pause without pressuring me."
**Avoiding over-interpretation**	Student treats the cue as a prompt, integrates verbal/contextual information and accepts ambiguity.	Student over-reads a single cue or lets cue monitoring dominate the consultation.	"The expression alone was ambiguous; asking what it meant was the safer response."

S18.3 Educator observation rubric for feed-forward

Domain	Illustrative 0–2 descriptor
**Notice**	0 = cue not noticed; 1 = noticed late/after repetition; 2 = noticed promptly without disrupting the consultation.
**Interpret tentatively**	0 = deterministic or unsupported label; 1 = partially qualified interpretation; 2 = clearly tentative and context-sensitive interpretation.
**Check directly**	0 = no check or leading assumption; 1 = closed/limited check; 2 = open question or teach-back that tests meaning.
**Adapt communication**	0 = no adaptation; 1 = generic or incomplete adaptation; 2 = response is matched to the checked concern or misunderstanding.
**Maintain consultation structure**	0 = cue focus displaces the clinical task; 1 = some loss of structure; 2 = cue response is integrated while history/explanation/safety-netting remains coherent.
**Reflect and recalibrate**	0 = unable to identify an alternative interpretation; 1 = identifies one alternative with prompting; 2 = independently identifies ambiguity, bias and a safer future response.

S18.4 Feedback sequence

Stage	Illustrative sequence
**First encounter**	1. SP completes the eight-item rating form. 2. Learner gives a brief self-assessment: one cue noticed, one uncertainty. 3. SP gives one behaviour-specific strength and one missed or ambiguous cue. 4. Educator identifies one feed-forward target for paired practice. 5. Learner states the phrase or behaviour they will try next.
**Final encounter**	1. SP completes the rating form before discussion. 2. Learner identifies what they noticed and how they checked it. 3. SP comments on attentiveness, clarity and the effect of any adaptation. 4. Educator calibrates interpretation, identifies over-reading or distraction where present, and reinforces the four-stage pathway. 5. Learner records one action for future simulated or clinical communication.

S18.5 Twenty-minute description–analysis–application debrief

Phase	Time	Facilitator purpose and prompts
**Opening and ground rule**	1 minute	"The aim is not to diagnose emotion from a face. We are examining how a possible cue can prompt respectful checking and communication adaptation."
**Description**	4 minutes	What did you notice? At what point? What did you say or do next? What did the SP do in response? Keep this stage descriptive rather than evaluative.
**Analysis**	8 minutes	What else might the cue have meant? Which contextual or verbal information supported or contradicted your first interpretation? Did your question check meaning or imply an answer? Did cue monitoring distract from the history or explanation? How might culture, neurodiversity, pain or communication style alter the cue?
**Application**	4 minutes	Which phrase will you use to acknowledge a cue tentatively? When will you rephrase, use teach-back or pause? How will you preserve consultation structure while remaining observant?
**Reflective writing launch**	3 minutes	Students begin the reflective journal, identifying one successful response, one missed or misinterpreted cue, and one concrete future action.

S18.6 Suggested facilitator sentence stems

- "What did you observe before you decided what it might mean?"
- "What other explanations could fit the same cue?"
- "How could you check that interpretation without labelling the patient?"
- "What changed in your communication after the patient answered?"
- "Did attending to the cue help or distract from the clinical task?"
- "What would you repeat, and what would you do differently next time?"

S18.7 Facilitator record

Field	Record
**Group/session code**	______________________________
**Facilitator(s)**	______________________________
**Main strength identified**	____
**Main calibration issue**	____
**Negative/discrepant example discussed**	____
**Agreed feed-forward action**	____
**Protocol deviation or safeguarding issue**	____

Supplementary Table S19: Participant-level confidence and recognition discordance

	At least one anxiety/confusion recognition outcome improved	Neither recognition outcome improved	Total
At least one primary confidence item improved	41	19	60
No primary confidence item improved	0	0	0
Total	41	19	60

Confidence improvement was defined as a positive change on Q1 or Q2. Recognition improvement was defined as a categorical transition from not meeting to meeting the criterion on anxiety or confusion recognition.

Supplementary Table S20. Comparison of MED with selected earlier programmes

Programme	Learners / design	Content and duration	Assessment	SP involvement	Reflection	Follow-up
Molinuevo et al. [19]	Second-year medical students; programme description rather than a controlled outcome evaluation.	Theoretical framework, learning settings, strategies, skills and assessment tools for decoding non-verbal clues; duration not reported in the published abstract.	Assessment tools and learner satisfaction described.	Not clearly reported.	Not clearly reported.	No longitudinal follow-up reported.
Ragsdale et al. [8]	156 healthcare providers and trainees; uncontrolled pre-post workshop at six US venues.	90-minute didactic session using progressively more difficult facial-expression examples followed by three interactive exercises.	Immediate skill, knowledge, perceived importance and confidence.	No standardised-patient encounter reported.	No structured reflective component reported.	No later follow-up reported.
Yu et al. [13]	82 first- and second-year medical students; randomised 41/41 to training or repeated-testing control.	One-hour class using METT and SETT.	Immediate METT and SETT recognition-test scores.	None reported.	None reported.	No later follow-up reported.
Park and Park [11]	51 first-year students analysed; single-cohort comparison before and after a broader communication course.	Five four-hour topic blocks (20 hours): one-hour lecture, two hours of SP practice and feedback, and one-hour instructor discussion for each topic.	Video-coded checklist of 12 non-verbal behaviours and a total score.	Repeated SP practice and formative feedback.	Instructor discussion; no formal reflective-journal component reported.	No later follow-up reported.
Kelly et al. [6]	112 conference participants attended; 73 completed evaluation; qualitative workshop evaluation across eight conferences.	Drama exercises addressing sight, sound, touch and proxemics; workshop duration not reported in the published summary.	Narrative workshop material and post-workshop open-ended questionnaire.	No standardised-patient encounter reported.	Experiential reflection was central to the workshop.	No later follow-up reported.
MED	60 final-year medical students; uncontrolled single-group convergent mixed-methods evaluation.	270 minutes: conceptual teaching, 14 teaching clips, first SP encounter, paired practice, final SP encounter, feedback, recognition task, debrief and journal.	Study-specific self-ratings, parallel-form recognition task, SP ratings and qualitative material.	Two different SPs per student; rating before feedback; SPs also provided feedback.	Structured debrief and reflective journal.	Immediate final assessment only; no follow-up.

Note: “Not reported” is used where a published report did not describe a component; it should not be interpreted as evidence that the component was absent.

Supplementary Table S21. Illustrative effect of multiplicity and clustering on the planned sample

Clustering source	Average cluster size	ICC 0.01	ICC 0.03	ICC 0.05	ICC 0.10
Teaching session	6	43	47	51	61
Final-encounter standardised patient	10	45	52	59	78
Medical school	20	49	64	80	118

Footnote: The independent-participant requirement was 40.576, rounded to 41, for a two-sided paired t-test with dz=0.50, 80% power and alpha=0.025. Each clustered value applies 1+(m-1)ICC to the unrounded requirement and rounds upwards. The calculations consider one clustering source at a time and are illustrative only. Teaching session, medical school and final-encounter standardised patient were considered separately as potential clustering sources; standardised patients were crossed with sessions, and only three schools were included.

Supplementary Table S22. Directionality of administered qualitative prompts and analytic handling

Source	Administered wording	Directional feature	Analytic handling
Student pre-training Q2	What do you hope to gain from this training programme?	Primes an expectation of benefit before teaching.	Used only to characterise expectations; not evidence of change.
Student post-training Q2	How has this training changed the way you approach patient interactions?	Presupposes that change occurred.	Coded as an elicited change account; not spontaneous confirmation.
Student post-training Q3	What strategies will you use to integrate micro-expression awareness into your future clinical practice?	Presupposes intended uptake and transfer.	Reported only as intended future use; no claim of actual transfer.
Student post-training Q4	Describe how recognising subtle emotional cues affected your communication during role-play encounters.	Presupposes recognition and an effect on communication.	Used as contextual explanation within simulation, not causal evidence.
SP open Q1	What did the student do particularly well during this interaction?	Solicits a positive account and does not invite criticism.	Not used to judge the balance of positive and negative experience.
SP open Q3	How did the student's attentiveness to your non-verbal cues affect your overall experience?	Presupposes attentiveness and an effect.	Treated as elicited context; not independent evidence of benefit.
SP open Q4	Did the student demonstrate empathy or clarification when you appeared uncertain or anxious?	Leading yes/no wording and introduces favourable constructs.	Not used to claim validated empathy or clinical competence.
Reflective prompt 1	Describe a specific moment ... when you successfully identified a patient's micro-expression.	Assumes successful recognition.	Interpreted as a prompted success example only.
Reflective prompt 3	Describe how recognising signs of anxiety or uncertainty affected the way you communicated...	Assumes recognition and an effect.	Context only; alternative and negative accounts actively sought.
Reflective prompt 4	Describe a moment when you changed or clarified your explanation because of...	Assumes that communication changed.	Used to illustrate the intended pathway, not to estimate prevalence of change.
Reflective prompt 5	How has this training changed your approach...	Presupposes change.	Coded as an elicited change account.
Reflective prompt 6	Did the training alter your understanding of empathy, attentiveness or rapport-building?	Suggests alteration and names favourable constructs.	Not used to support a claim that empathy improved.
Reflective prompt 8	Suggest strategies for integrating cue awareness ... to improve communication.	Assumes potential improvement.	Treated as suggestions, not observed benefit.
Reflective prompt 9	In what types of consultations do you think these skills will be most useful...?	Assumes usefulness.	Reported only as perceived future relevance.
Reflective prompt 10	Reflect on how this training may influence your future communication style...	Assumes future influence.	Reported only as intention.

Note: Prompt source should remain attached to each coded extract. Favourable responses to directional prompts are interpreted as elicited accounts, while neutral, negative and discrepant material is actively sought across the corpus.

Supplementary Table S23. Reporting-framework map

Framework	Role in this paper	Main reporting domains	Principal locations
DoCTRINE [28]	Overarching education-specific framework for a health-professions educational innovation.	Problem and rationale; curriculum development; implementation; outcomes; interpretation in relation to prior educational work.	Introduction; Sections 2.3-2.5; Results; Discussion; Tables 1 and S20.
STROBE [24]	Relevant elements of the uncontrolled observational quantitative strand; not presented as a complete design match.	Design, setting, participants, variables, missing data, statistical methods, estimates and limitations.	Sections 2.1-2.2, 2.6, 2.8 and 3.1-3.4; Tables 2-4.
TIDieR [25]	Reproducible description of the intervention and delivery.	Rationale, materials, providers, procedures, mode, setting, schedule, modifications and fidelity.	Sections 2.3-2.5 and 3.2; Table 1; Tables S5, S7 and S16; Instruments S17-S18.
GRAMMS [26]	Rationale and reporting of the convergent mixed-methods design.	Reason for mixing, timing and priority, separate analyses, integration, discordance and mixed-methods limitations.	Sections 2.1, 2.7, 3.5-3.6 and Discussion; Table 6; Tables S19 and S22.

Footnote: The frameworks informed reporting. The manuscript should not claim complete compliance unless the corresponding checklist has been completed and checked.

Supplementary Figure S1: Individual change scores. Change was calculated as the immediate-final score minus the initial score. For Q1 and Q2, the initial score was obtained before teaching; for SP1 and SP8, the initial score was obtained during the first pre-practice simulated encounter.

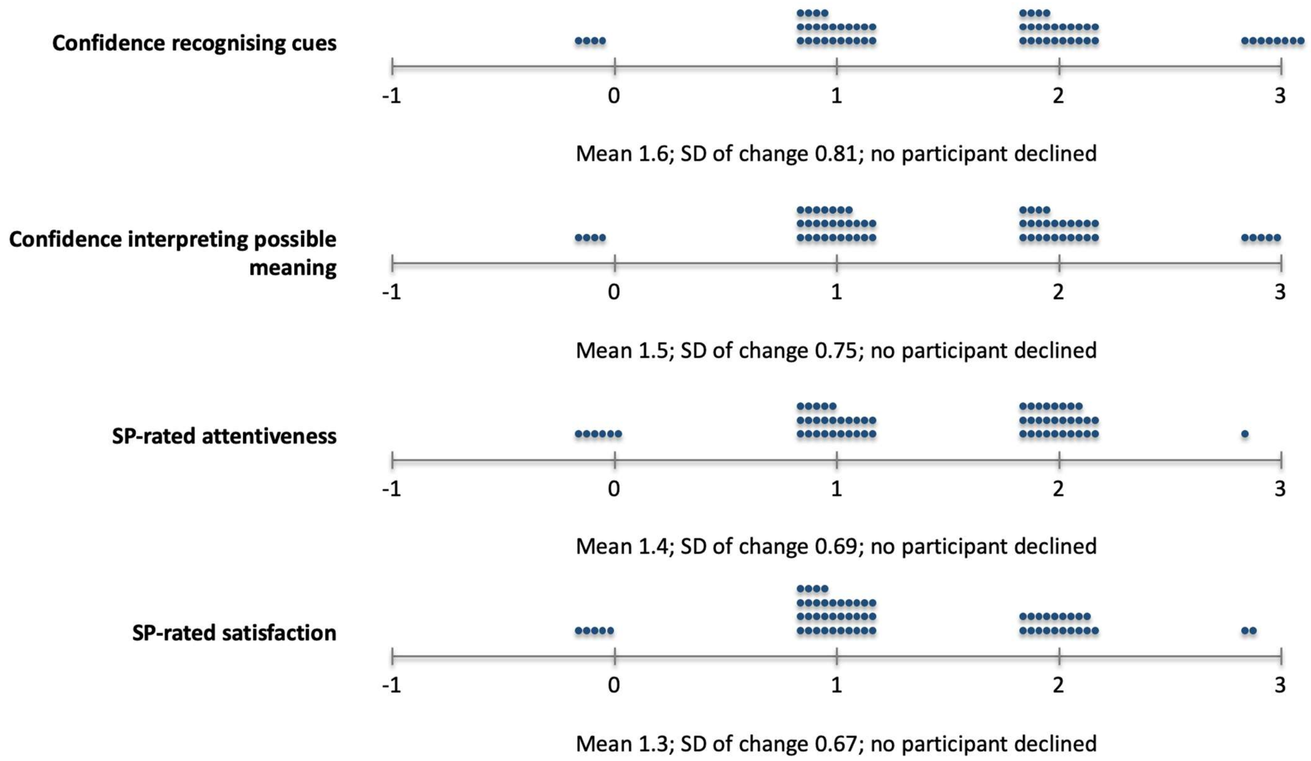

Supplement: Supplementary file 1 [file clinpract-16-00170-s001.zip › clinpract-4341941-supplementary.pdf]
